# An Integrated Multi-Media Modeling System for Regional- to National-Scale Nitrogen and Crop Productivity Assessments

**DOI:** 10.3390/agriculture15101017

**Published:** 2025-05-08

**Authors:** Yongping Yuan, Xiuying Wang, Verel Benson, Limei Ran

**Affiliations:** 1 Office of Research and Development, U.S. Environmental Protection Agency, Research Triangle Park, NC 27711, USA; 2 AGORO Carbon Alliance, Lincoln, NE 68512, USA; 3 Benson Consulting, Columbia, MO 65203, USA; 4 Soil Science and Resource Assessment, Natural Resources Conservation Service Resource, U.S. Department of Agriculture, Raleigh, NC 27609, USA

**Keywords:** fertilizer management scenario, crop yield, nitrogen loss, IMMMS

## Abstract

Excessive nutrients transported from agricultural fields into the environment are causing environmental and ecological problems. This study uses an integrated multi-media modeling system version 1 (IMMMS 1.0) linking air, land surface, and watershed processes to assess corn grain yield and nitrogen (N) losses resulting from changing fertilization conditions across the contiguous United States. Two fertilizer management scenarios (FMSs) were compared and evaluated: 2006 FMS, developed based on the 2006 fertilizer sales data; and 2011 FMS, developed based on 2011 fertilizer sales and manure. Corn grain yields captured historical reported values with average percent errors of 4.8% and 0.7% for the 2006 FMS and 2011 FMS, respectively. Increased nitrogen (N) application of 21.2% resulted in a slightly increased corn grain yield of 5% in the 2011 FMS, but the simulated total N loss (through denitrification, volatilization, water, and sediment) increased to 49.3%. A better correlation was identified between crop N uptake and N application in the 2006 FMS (R^2^ = 0.60) than the 2011 FMS (R^2^ = 0.51), indicating that applied N was better utilized by crops in the 2006 FMS. Animal manure could create nutrient surpluses and lead to greater N loss, as identified in the regions of the Pacific and Southern Plains in the 2011 FMS. Manure nutrient management is important and urgently needed to protect our air and water quality. The IMMMS 1.0 is responsive to different FMSs and can be utilized to address alternative management scenarios to determine their impact when addressing the sustainability of food production and environmental issues.

## 1. Introduction

Human population growth and increased demands for food, energy, and transportation have led to dramatic increases in nitrogen (N) demand and production [1,2]. Synthetic N fertilizer production has converted a large amount of unreactive N to reactive N [3], and agricultural fertilizer use is the largest consumer of terrestrial reactive N [4]. Although agricultural fertilization is essential for plant growth, enhancing cropland productivity, and sustaining food production, excessive and imbalanced fertilizer use has dramatically altered the global nutrient budget and caused unintended adverse environmental and ecological problems, including soil acidification and degradation, groundwater contamination, eutrophication of fresh water and marine ecosystems, hypoxia, biodiversity loss, and impaired atmosphere related to emissions of N oxides, ammonia gas, and the accumulation of nitrous oxide [4–12].

**Figure 1.**
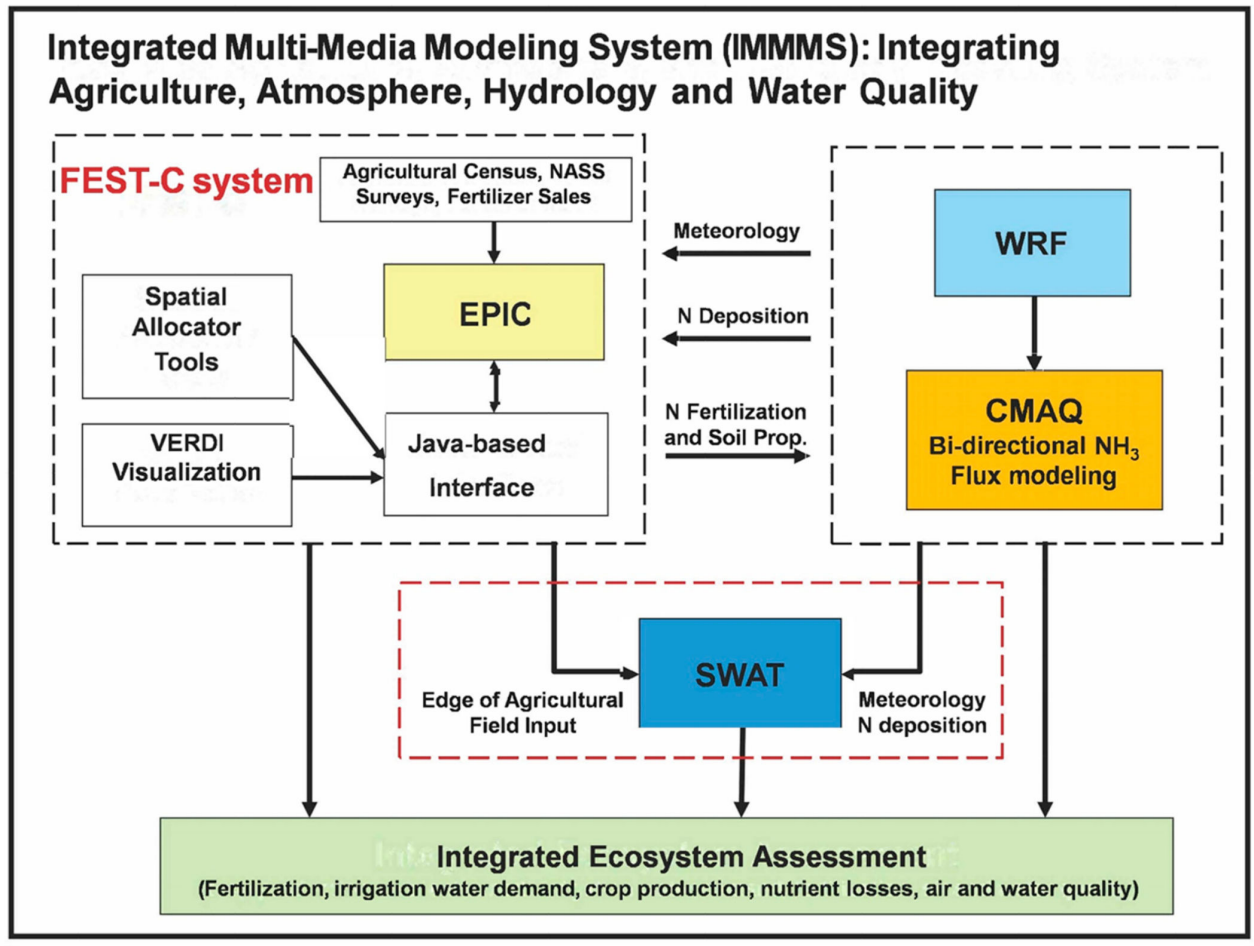
The IMMMS 1.0. NASS: National Agricultural Statistics Service; FEST-C: Fertilizer Emission Scenario Tool for CMAQ; CMAQ: Community Multi-Scale Air Quality; EPIC: Environmental Policy Integrated Climate; WRF: Water Research and Forecasting; SWAT: Soil and Water Assessment Tool.

When facing the three challenges of food security, environmental degradation, and climate variation, fertilizer management strategies such as improving N use efficiency (NUE), which is the ratio of the N used by plants to the N applied, have been widely studied for reducing N losses from agricultural fields for sustainable development [11,13–15]. In addition, due to the complex N cycle and its dynamics from the atmosphere to the biosphere, through N dry deposition and wet deposition, the U.S. Environmental Protection Agency (USEPA) Science Advisory Board [16] and the European Nitrogen Assessment [17] emphasized the need for integrated, multi-media and transdisciplinary approaches to comprehensively evaluate the fate and transport of N. Therefore, the USEPA has developed the integrated multi-media modeling system version 1 (IMMMS 1.0) [18] by combining the Soil and Water Assessment Tool (SWAT) with the previously developed Fertilizer Emission Scenario Tool for Community Multi-Scale Air Quality (CMAQ) (FEST-C) system [19] (Figure 1). More information about the development of IMMMS 1.0 can be found in the Supplementary Materials. Although the capability of the IMMMS 1.0 to link air, land surface, and watershed processes to address impacts of fertilization and meteorology on crop production, N losses, and air and water quality has been established, the system needs to be thoroughly evaluated on various components and continuously enhanced to incorporate the latest science and data to ensure it stays robust and meets ongoing environmental challenges.

Fertilization is one of the key inputs in simulating crop production and N losses. The existing FEST-C version 1.4, available at (https://www.cmascenter.org/fest-c/ (accessed on 19 April 2025)), was established with two fertilizer management scenarios (FMSs) (2001 and 2006). Both scenarios were based on information from the U.S. National Agricultural Statistics Service (NASS), Conservation Effects Assessment Project (CEAP), and fertilizer sales data for different agricultural production regions across the contiguous United States (CONUS). Those data were released about every five years; thus, data released in 2011 and later were not considered during the early development of the modeling system. As a result, there was a need to incorporate data released in 2011 and beyond to represent conditions in more recent years.

The 2006 FMS was used in all previous applications [18–21]. The focus of Ellen et al.’s study was linking the Environmental Policy Integrated Climate (EPIC) model for estimating ammonia (NH_3_) emissions from the application of inorganic nitrogen fertilizers to agricultural soils [19] for CMAQ. The focus of Yuan et al.’s study was the integration of SWAT and EPIC, IMMMS 1.0, and its application on the Mississippi River Basin [18]. The focus of Pleim et al.’s study was to validate ammonia (NH_3_) emissions by comparing modeling results with measurements [20]. More details on these studies can be found in the Supplementary Materials. In the 2006 FMS development, only fertilizer sale data were used [19]. We developed the 2011 FMS based on 2011 fertilizer sale data and animal manure from the Confined Animal Facility Operation (CAFO) (see Supplementary Materials). However, evaluations of the newly added 2011 FMS have not been conducted. In addition, the U.S.’s corn production is the largest in the world, with operations concentrated in the Midwest (Corn Belt). Nitrogen application is one of the key factors to boost corn yield. Thus, the evaluation of simulated corn yield with NASS reported values comprised an important demonstration of the modeling system. Furthermore, EPIC was simulated during individual years rather than continuously over multiple years due to limitations in the system’s initial construction. The current FEST-C version 1.4 has been upgraded to make continuous simulations on a daily basis. Therefore, the overall objective of this study was to compare and evaluate the two FMSs (2006 and 2011) with a focus on assessing the regional-to-national response of spatially explicit crop-specific N losses and corn grain yield to changing fertilization conditions across CONUS.

## 2. Materials and Methods

### 2.1. Brief Introduction of the Integrated Multi-Media Modeling System

The integrated multi-media modeling system (IMMMS 1.0) includes the following models: (1) CMAQ, (2) Water Research and Forecasting (WRF), (3) EPIC, (4) SWAT, and the Java-based user interface FEST-C (Figure 1). Yuan et al. (2018) provided detailed descriptions of each of these models, the FEST-C interface, and the IMMMS 1.0 [18]. Briefly, the Java-based user interface FEST-C facilitates the integration of various models and guides users through the following: generating land use and crop data needed for EPIC; creating daily weather and N deposition input from WRF/CMAQ; preparing EPIC site, soil, and management inputs (Spatial Allocator Tools in Figure 1) for EPIC simulations; and extracting EPIC output for quality assurance and for required SWAT inputs. In addition, FEST-C also extracts initial soil and pH conditions and the daily N information required for CMAQ bi-directional NH_3_ modeling.

The CMAQ version employs a compensation point approach to estimate the flux of NH_3_ (emission or deposition) from underlying soil and vegetated surfaces to air [19]. EPIC was modified to take daily time series measurements of total wet oxidized N (g/ha), total wet reduced N (g/ha), total dry oxidized N (g/ha), total dry reduced N (g/ha), and total wet organic N (g/ha) from WRF/CMAQ [19].

The core EPIC is a comprehensive terrestrial ecosystem model capable of simulating the farming operations used to grow crops, such as planting, harvesting, fertilization, tillage operation, irrigation, hydrology, carbon (C) and nutrient cycling, and dynamic soil biogeochemical properties under various management practices and soil conditions. Crop residue remaining on the field after harvest is transformed into organic matter. This organic matter may build up in the soil over time, or it may degrade, depending on climatic conditions, cropping systems, and management. The model dynamically represents multiple soil properties, including the depth and bulk density for each soil layer during simulations. The model removes eroded soil, attached organic nutrients, pesticides, and C from the soil profile as part of the emphasis on erosion productivity [22,23]. EPIC has been widely used to assess soil erosion, crop productivity, irrigation, climate change, soil organic C, and nutrient losses [24,25].

SWAT [26–28] has been widely applied to evaluate best management practices, alternative land use/land management, and climate change on pollutant losses to streams within a watershed [27,29–32]. Integrating SWAT with the CMAQ/WRF/EPIC improved SWAT simulation results, as it incorporates more detailed field-scale biogeochemical processes by using EPIC for agricultural land simulations; on the other hand, integrating SWAT with CMAQ/WRF/EPIC, as carried out by the IMMMS 1.0, allows for its use within large river basins because stream/channel processes can be simulated after integrating the widely used watershed model. The integration strengthens the assessment of the impacts of future climate scenarios, regulatory and voluntary programs for N oxide air emissions, and land use and land management on N transport and transformation in large river basins [18].

### 2.2. Model Inputs and Configuration

Detailed information on the initial construction of the FEST-C system and EPIC input configuration, including crops, crop management, soil information, and weather, can be found in Cooter et al. [19] and the Supplementary Materials. Since the system was set up to represent the contiguous United States (CONUS) at a 12 km resolution, essential inputs were developed and stored as common data to facilitate the integration of EPIC with WRF/CMAQ and make the system more user-friendly. For example, soil files needed to run the system were stored as common data in the system. For crops and crop management, the National Land Cover Database (NLCD) and USDA NASS Census of Agriculture (COA) were used, and fractions of crop land in each simulation grid (12 by 12 km) were assigned based on the COA county-level spatial crop assignment (see the Supplementary Materials for more details). Fertilizer application variables such as timing and amount are essential components of crop management. Although our goal is to be as spatially explicit as possible, it is impossible to capture daily farming activities such as when, what type, and how much of the N fertilizers were applied at such a spatial scale (reginal to national). Therefore, FMSs were developed. The goal of developing FMSs is to facilitate the characterization of broad trends in current and future crop management and fertilizer application practices that are likely to affect air quality and atmospheric deposition, crop yields, and N losses on regional to national scales, rather than targeting behaviors of a specific, potentially unique fertilizer application scenario that might have only a limited spatial scale of influence. Thus, we tried to automate this process to the greatest extent possible.

Essentially, EPIC was set to trigger auto-N applications when plants suffered a given level of N stress, and a fixed auto-N application rate was set to a percentage of the annual N applied at the modeling grid. For each 12 km by 12 km grid, the amount of N initially applied was a fixed fraction of an annual EPIC 5 yr average amount, but the date of application varied with crop, crop variety, local soil, and weather conditions, leading to more spatially and temporally resolved application estimates [19].

During the 2006 FMS configuration, 2006 NLCD and 2007 NASS COA were used. The U.S. NASS fertilizer sale data were allocated for crop use, and N from animal manure was not considered. However, when crop nutrient demand exceeded inorganic fertilizer N sales, the shortfalls from crop demand were assumed to be met with animal manure [19]. The development of the 2011 FMS followed the same methodology. This design was to create patterns of N uses that would be connected to the spin-up estimates of crop needs under different soil and weather conditions. In the 2011 configuration, the 2011 NLCD and 2012 NASS COA were used. The U.S. NASS data on fertilizer sales and nutrients from animal manure were applied to the FMS. More details on the FMS development can be found in the Supplementary Materials.

### 2.3. Model Simulations

The Java-based user interface FEST-C can be used to facilitate model simulations. The user manual is available at https://www.cmascenter.org/fest-c/ (accessed on 19 April 2025). In setting up simulations, a user can make selections through the user interface. For example, the system can be run using the 2006 FMS or 2011 FMS. For this analysis, the model was set up to run for corn grain grown in 12 km domain grids over the CONUS from 2003 to 2017 continuously with the 2006 FMS first, then again with the 2011 FMS. For both FMSs, although fertilizer types, timings, and allocation fractions for each of the USDA agricultural production regions (Figure S1) were based on fertilizer sales over six-month periods, specific application rates and dates for each modeled grid were estimated by EPIC based on spin-up runs. The annual plant N need from the last 5 years of spin-up runs for corn grain, together with total available nutrient for crop consumption, were used to calculate application rates. There was no attempt to vary the type of fertilizer below a regional level because there is no consistent source of information to estimate this variability at the 12 km grid level over the CONUS. Considering farmers do vary their fertilizer rates to meet their objective, EPIC was set to trigger auto-N application when plants suffered 40% or more of N stress. The fixed auto-N application rate was set to 30% of the annual elemental N applied at the modeling grid based on the last 5 years of the spin-up average amount. Minimal time between applications was set to 7 days, and maximum annual N fertilizer application for a crop was set to 300 kg N ha^−1^.

Rainfed and irrigated corn grain lands were simulated separately. An EPIC automatic irrigation schedule triggered by soil–water tension at 100 kPa was used. Sprinkler irrigation was assumed, with a single application ranging from 25 to 35 mm and a maximum annual irrigation volume of 2000 mm. Crop management was created from the USDA NASS Agricultural Resource Management Survey data, and tillage operation was designed to represent conservation tillage.

### 2.4. Model Evaluation of Corn Grain Yield, N Losses and NUE

After the simulation of both rainfed and irrigated cultivations, aggregated outputs from both cultivation systems were calculated for each grid using area-weight averaging. Firstly, we compared N applications between the 2006 FMS and 2011 FMS. Secondly, we compared domain-wide corn grain yields weighted by area between the 2006 and 2011 FMSs. In addition, corn grain yields were also evaluated by comparing simulated to historical reported crop grain yield from USDA NASS [33]. Thirdly, N losses from both FMSs were evaluated. We performed regression analysis for N uptake and N loss between two FMSs. Finally, we looked at crop uptake and NUE to gain insights on N application and crop production.

## 3. Results and Discussion

### 3.1. Comparison of N Application Between 2006 FMS and 2011 FMS

As described in the introduction, all previous applications of this modeling system used the 2006 FMS [18,19,21,23]. Cooter et al. [19] evaluated the 5-year average annual crop-based estimates of inorganic N use (N_fer_) and concluded that the N_fer_ amounts agreed well with reported spatial patterns produced by others. Thus, in this study, we compared N_fer_ from the 2011 FMS with N_fer_ from 2006 FMS.

The dominant external N input to corn grain production was inorganic commercial fertilizer (N_fer_), with N_fer_ rates averaging at 145.9 and 144.5 kg N ha^−1^ yr^−1^ over the CONUS in the 2006 and 2011 FMSs, respectively (Table 1). As described in the 2006 FMS configuration, when crop nutrient demand exceeded inorganic fertilizer N sales, the shortfalls from crop demand were assumed to be met with N from animal manure (N_man_). Thus, only a portion of animal manure was used in the 2006 FMS, with N_man_ averaged at 6.8 kg N ha^−1^ yr^−1^. The 2011 FMS included livestock manure from CAFO, which resulted in an average of 40.6 kg N ha^−1^ yr^−1^ (Table 1).

**Figure 2.**
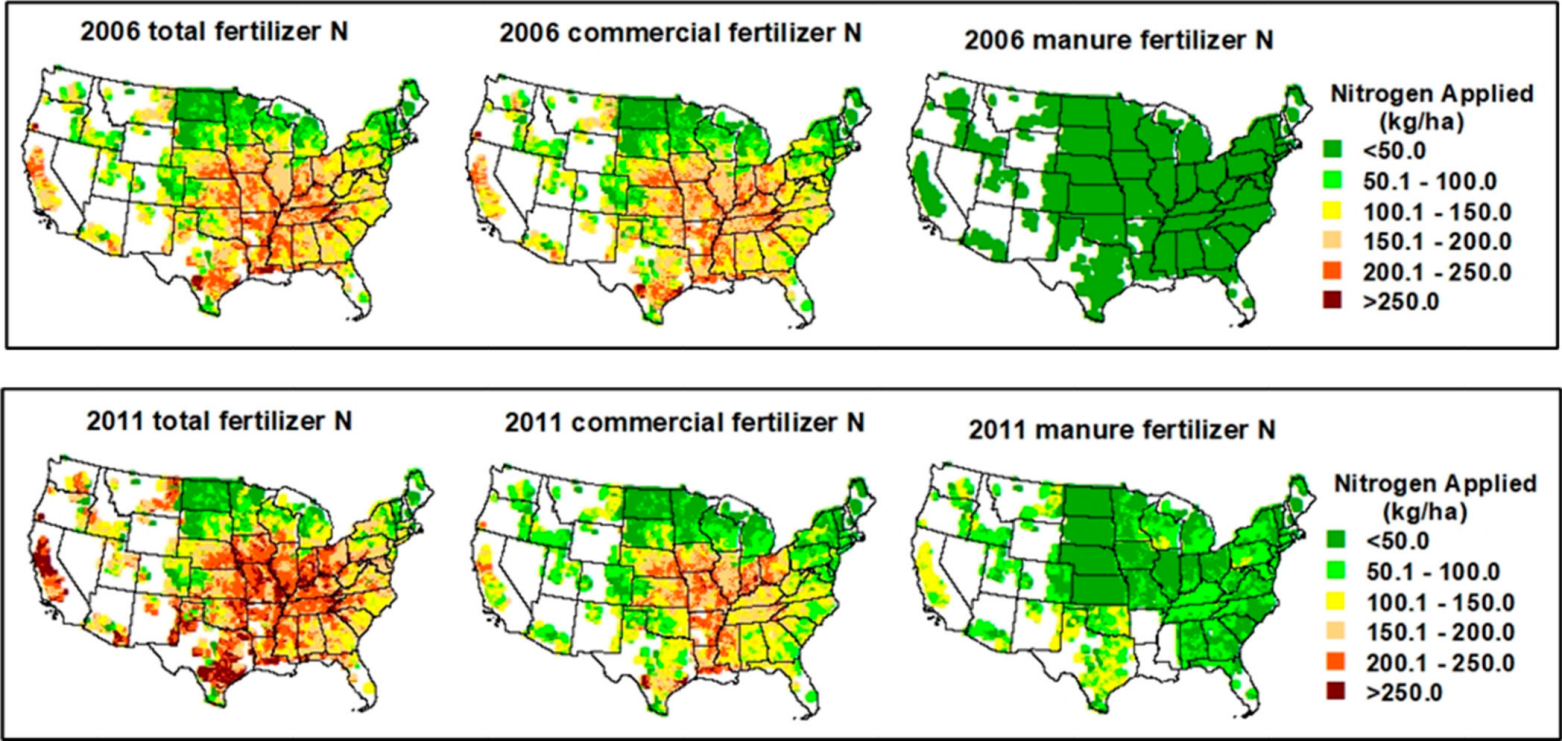
Annual average total, commercial, and manure fertilizer N application rates in model simulations for the 2006 and 2011 fertilizer management scenarios (FMSs) over the contiguous United States (CONUS) 12 km domain grids with corn grain land use.

The highest N application (N_app_), 217.6 kg N ha^−1^ yr^−1^ in 2006 FMS and 225.8 kg N ha^−1^ yr^−1^ in the 2011 FMS, respectively, occurred in the Delta States (Table 1). Comparing the 2006 FMS and 2011 FMS, there was no manure applied in this region in the 2011 FMS (Figure 2) based on the Ag Census data; the N_man_ of 6.1 kg N ha^−1^ yr^−1^ from the 2006 FMS (Table 1) revealed limitations of the 2006 FMS configuration since animal manure may not be available in the region. In addition, the Corn Belt and Northern Plains regions, which account for 70% of the total corn grain area, have a higher N application rate from commercial fertilizers in the 2011 FMS than in the 2006 FMS. The remaining regions have lower N application rates from commercial fertilizers in 2011 than in 2006. Therefore, the average N application rate from commercial fertilizers over the CONUS in the 2011 FMS was slightly lower (by 0.9%, Table 1). However, since the total N application is the combination of commercial fertilizer and manure N sources, the total N application is higher in 2011 than that in 2006 due to N sources from manure in the 2011 FMS. The average annual N application is higher in all the 10 regions, with an average of 21% higher over the CONUS (Table 1) in 2011 than that in 2006.

Fertilizer management employed in the cultivated cropland was mainly to maximize crop yield production. Nitrogen (N) sources contributed to corn production include external sources and internal sources, which is N from mineralization. Due to the difference in availability of fertilizers between the 2006 FMS and 2011 FMS, as well as the different soils and climate conditions across the CONUS, the actual fertilizers used by corn production were different spatially and temporarily. The annual average N application (N_app_), a combination of commercial and animal manure, shows variations throughout the corn grain areas mainly due to different soils and climate conditions across the CONUS (Figure 2). Figure 2 also demonstrates spatial dynamics associated with N demand, which is also impacted by soil and weather conditions. Furthermore, in some places, the application rates show differences between neighboring states, revealing the state-level nature of the fertilizer sales information from USDA NASS. As expected, the areas of intensive animal agriculture can be better identified in the 2011 manure fertilizer map (e.g., higher application rates such as parts of Texas and California).

### 3.2. Corn Grain Yield Response to 2006 FMS and 2011 FMS

**Figure 3.**
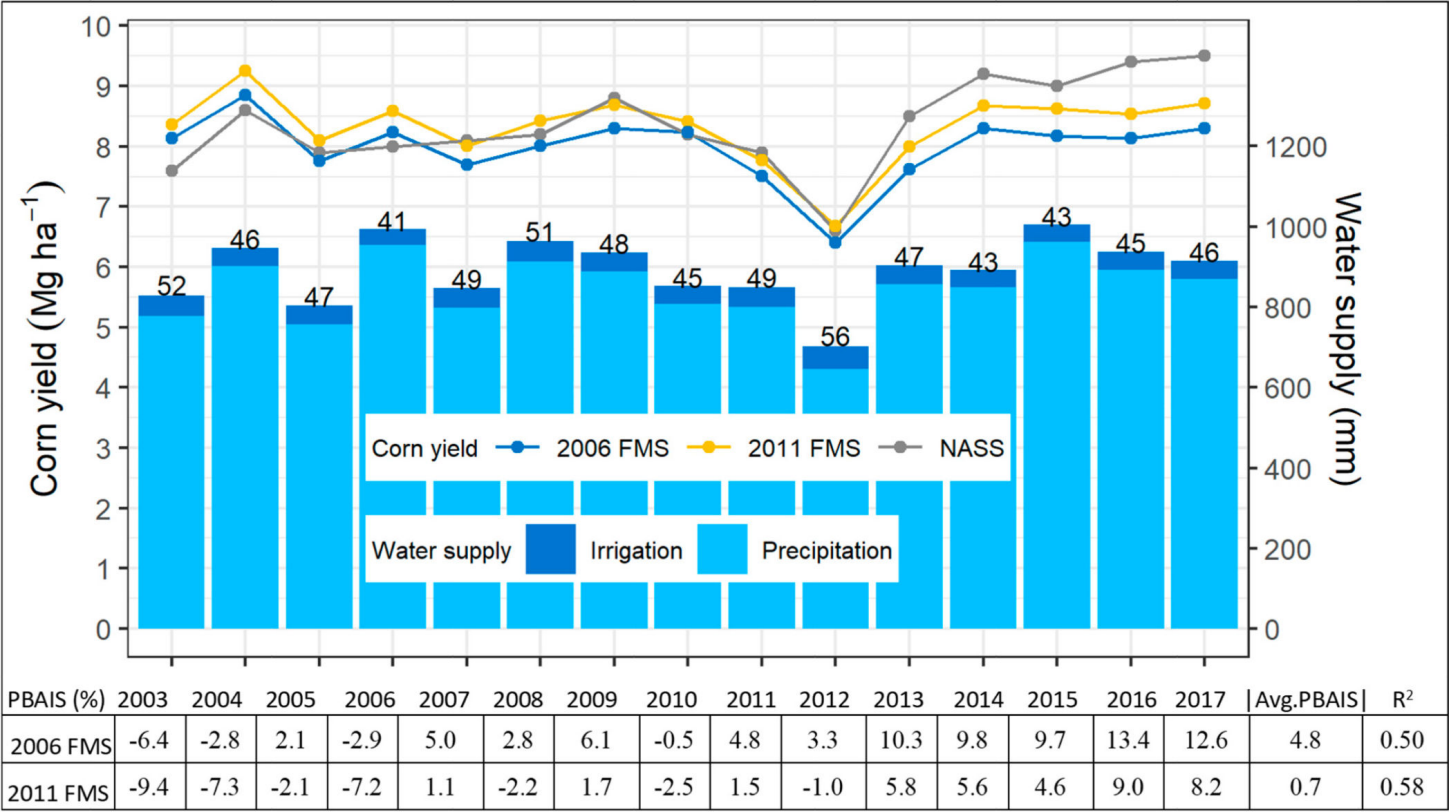
Simulated corn grain yield (domain wide) in comparison with USDA NASS: National Agricultural Statistics Service (NASS); reports for the 2006 and 2011 FMSs. Note: area-weighted values of irrigation amount in mm are labeled.

Compared with the reported corn grain yield (2003 to 2017) from USDA NASS, both simulated yields followed the reported trend reasonably well (Figure 3), with R^2^ values of 0.50 and 0.58 for the 2006 and 2011 FMSs, respectively. The percentage errors ranged from −13.4% to 6.4% in the 2006 FMS, with average annual error of 4.8%, while the percentage errors ranged from −9.4% to 9.0% in the 2011 FMS, with average annual error of 0.7% (Figure 3). Variations in corn grain yields from year to year (Figure 3) partially reflected variability in weather conditions. For example, the driest year (2012) had the lowest yields (NASS reported as well as simulated; also see precipitation and irrigation in Figure 3). As expected, fertilizer inputs also played a key role in crop yields, and higher N_app_ resulted in higher yields in general, as demonstrated by the different crop yields simulated for the two FMSs (Figure 3 and Table 2). Corn grain yield was slightly higher for the FMS 2011 due to higher N_app_ across the production regions (∆N_app_ from 3.8% to 40.6%, Table 1), mainly from the inclusion of N_man_ in the 2011 FMS. After the 2012 dry year, the NASS reported yields were consistently higher than simulated yields for both FMSs, leading to under-prediction from 2013 to 2017. The 2011 FMS did a better job representing the NASS yield report than the 2006 FMS in these later years, which may also be due to the inclusion of manure in the 2011 FMS. Inclusion of manure nutrient increased the nutrient availability to crop uptake, which in turn increased the potential for higher crop yield.

Comparing the N use efficiency (NUE) from the 2006 FMS and 2011 FMS, NUE values ranged from 0.51 to 0.90 in the 2006 FMS, with an area-weighted value of 0.65 over all regions. In the 2011 FMS, NUE values ranged from 0.43 to 0.73, and the area-weighted value was 0.56 (Table 2). The regional differences in NUE values over the CONUS illustrate the impact of a complex set of factors, including soil properties, climate, and fertilizer management intensity on NUE. For North America, Fixen et al. [34] reported an average NUE value of 0.68 for cereals (primarily corn, rice, and wheat) based on crop yields and associated average fertilizer N rates, which did not include manure N (thus, NUE would be lower if manure N inputs were included).

The highest NUE values (Table 2) were in the Lake States for both FMSs, corresponding to the lowest N_app_ rates of 97.6 and 126.2 kg N ha^−a1^ in the two FMSs (Table 1), respectively. The lowest NUE values were associated with the highest N_app_ rates of 217.6 (Delta States in 2006) and 239.7 (Southern Plains in 2011) kg N ha^−1^ in the two FMSs, respectively. As demonstrated in previous studies [15,35], corn NUE declined in response to a high level of N input rates, particularly in the intensive corn producing states (e.g., Corn Belt states). Although the application of N-based fertilizer is essential to maintaining high crop yield, lower levels of NUE values corresponding to higher N_app_ rates indicate greater potential for nutrient losses to the environment, especially in the major corn production regions (e.g., Corn Belt and Southern Plains).

### 3.3. N Losses from 2006 FMS and 2011 FMS

Nitrogen (N) losses from cultivated croplands depend on external N inputs (fertilization, atmospheric N deposition, and fixation), crop N uptake and harvest, volatilization, runoff and erosion (affected by soil properties, crop cover, and climatic conditions), and the dynamics and transformation of N in soil. The IMMMS 1.0 linking atmosphere, agriculture, and hydrologic processes provides a unique opportunity to address N issues of air pollution and water quality associated with agricultural production.

**Figure 4.**
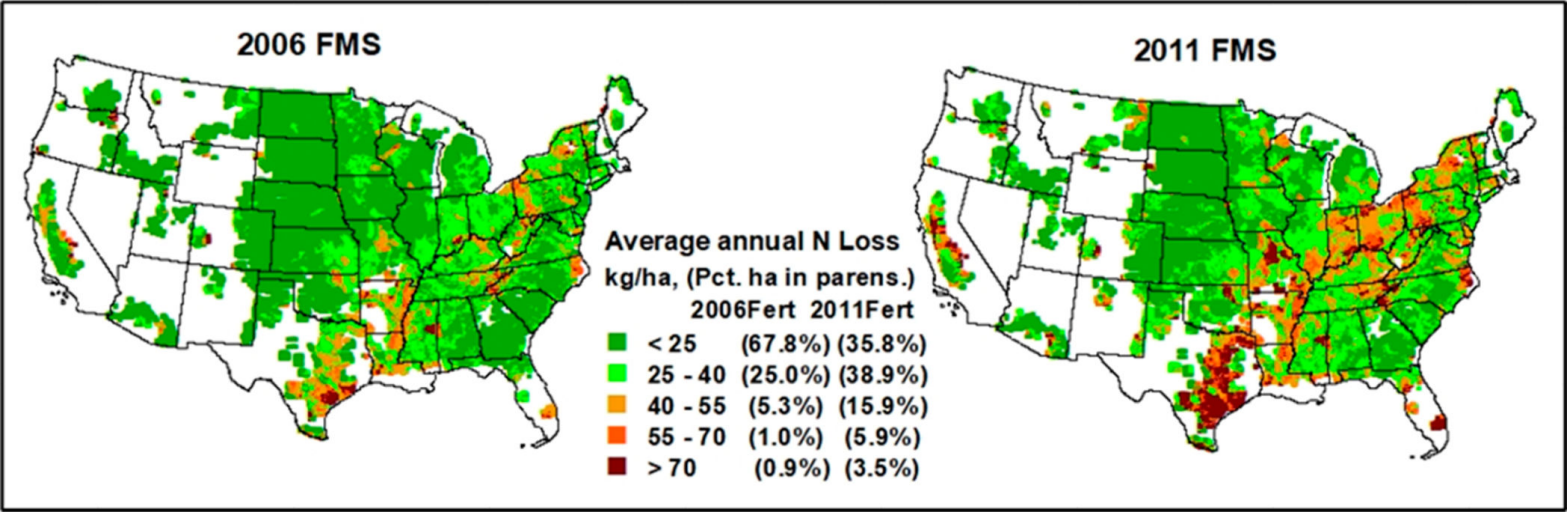
Average annual per-hectare N_loss_ from corn grain area simulated for the 2006 and 2011 FMSs over the contiguous United States (CONUS) 12 km domain grids during the simulation period from 2003 to 2017.

Nitrogen loss (N_loss_) from all pathways over the CONUS domain grid illustrates the variation across the CONUS (Figure 4). The highest N losses are colored dark red and red. Comparing the 2006 FMS with the 2011 FMS, 1.9% and 9.4% of the corn grain area in the 2006 FMS and 2011 FMS, respectively, had N_loss_ above 55 kg N ha^−1^ yr^−1^ (e.g., southeastern Texas, Indiana, and Pennsylvania). About 30.3% (2006 FMS) and 54.8% (2011 FMS) of the area had N_loss_ between 25 and 55 N ha^−1^ yr^−1^. The areas with the least N loss, represented in green on the map, comprised 67.8% (2006 FMS) and 35.8% (2011 FMS) of the corn grain area and had N_loss_ rates below 25 kg N ha^−1^ y^−1^ on average over the simulation period from 2003 to 2017.

Nitrogen loss (N_loss_) pathways include the following: loss through denitrification (N_den_), loss through surface runoff and/or leaching (N_wat_), loss through vitalization (N_vol_), and loss through soil erosion and sediment transport (N_sed_) (Table 3). In the 2006 FMS, N_loss_ ranged from 9.9 to 46.1 kg N ha^−1^ yr^−1^ across the ten production regions, and the average N_loss_ at the national level was 21.9 kg N ha^−1^ (Table 3), which accounts for about 14% of N_app_ (N_app_ was 152.7 kg N ha^−1^ in Table 1). The highest loss was through vitalization (N_vol_), followed by N_wat_ and N_den_ (Table 3). In the 2011 FMS, the N_loss_ ranged from 17.8 to 61.8 kg N ha^−1^ yr^−1^ across the ten production regions, with N_loss_ of 32.7 kg N ha^−1^ yr^−1^, accounting for about 18% of N_app_ at the national level (N_app_ was 185.1 kg N ha^−1^ in Table 1). The highest loss was through N denitrification (N_den_), followed by N_vol_ and N_wat_ (Table 3). The least N loss was through soil erosion and sediment in both FMSs. At the regional level, about 10% to 24% of N_app_ was lost, as estimated in the 2006 FMS, and about 12% to 26% of N_app_ was lost in the 2011 FMS (Table 3). A relatively low N loss to soil erosion and sediment, which resulted in overall lower N loss ratios (N_loss_/N_app_), was a result of conservation tillage. Long promoted as a key management practice for enhancing soil quality and further reducing soil erosion, conservation tillage is widely used across the nation [36–40].

The Natural Resources Conservation Service [40] reported that N_vol_ averaged 7.3 kg N ha^−1^ yr^−1^ from all cultivated cropland at the national level. In this study, the EPIC-estimated N_vol_ rates ranged from 4.4 to 9.2 kg N ha^−1^ yr^−1^ in the 2006 FMS and from 6.6 to 14.6 kg N ha^−1^ yr^−1^ in the 2011 FMS for corn grain over the CONUS. Denitrification (N_den_) had high spatial and temporal variability in most ecosystems and was impacted by temperature, soil–water content, and soil C and N contents [41]. N_den_ rates can be on the order of 20–50 kg N ha^−1^ yr^−1^ and range from 5% to 20% of N_app_ [41]. In this study, N_den_ rates ranged from 1.7 to 25.0 kg N ha^−1^ yr^−1^ in the 2006 FMS and from 6.4 to 41.7 kg N ha^−1^ yr^−1^ in the 2011 FMS (Table 3) among the 10 production regions. The area-weighted N_den_ rate over the CONUS was 3.6% of N_app_ in the 2006 FMS and 5.8% of N_app_ in the 2011 FMS. The magnitude of EPIC-simulated N_vol_ and N_den_ rates in this study is reasonable.

**Figure 5.**
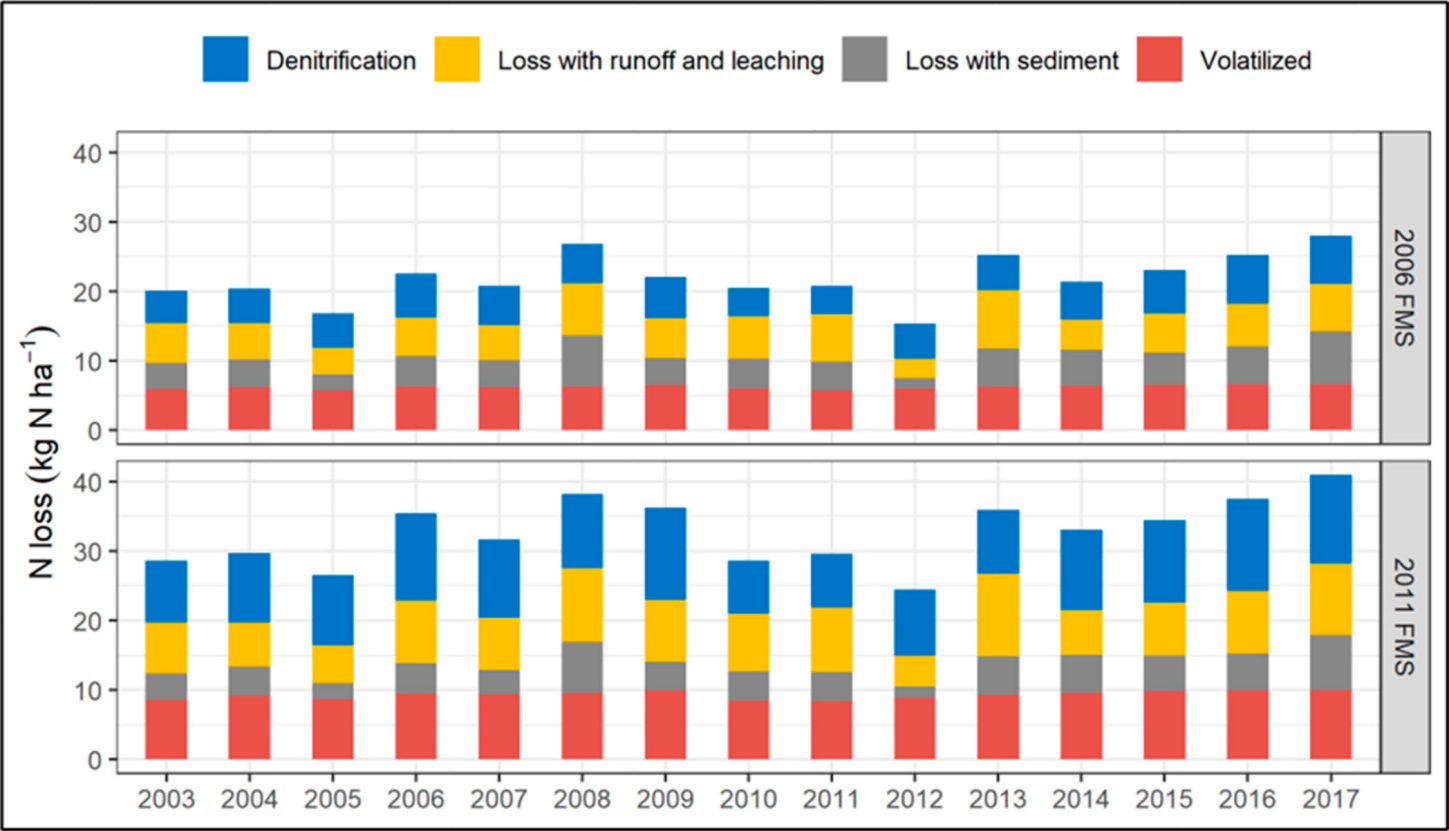
Area-weighted yearly N losses for corn grain over the CONUS for the 2006 and 2011 FMSs.

Across the CONUS, annual N losses varied year to year (Figure 5) mainly due to variations in N inputs and weather conditions. Higher N losses were observed for the 2011 FMS than the 2006 FMS for all years. Within the simulation period from 2003 to 2017, the highest per-hectare losses occurred in 2017, associated with higher N inputs in the two FMSs, respectively. N_loss_ rates averaged 28 and 41 kg N ha^−1^ yr^−1^ in the two FMSs over the simulation period, respectively. The lowest per-hectare losses occurred in 2012 (the driest year during the simulation period, averaging 646 mm precipitation) in both FMSs, with N_loss_ values of 15.4 and 24.4 kg N ha^−1^ yr^−1^, respectively (Figure 5).

### 3.4. Connecting Nitrogen Inputs, Plant Uptakes and Crop Yields, and N Losses

**Figure 6.**
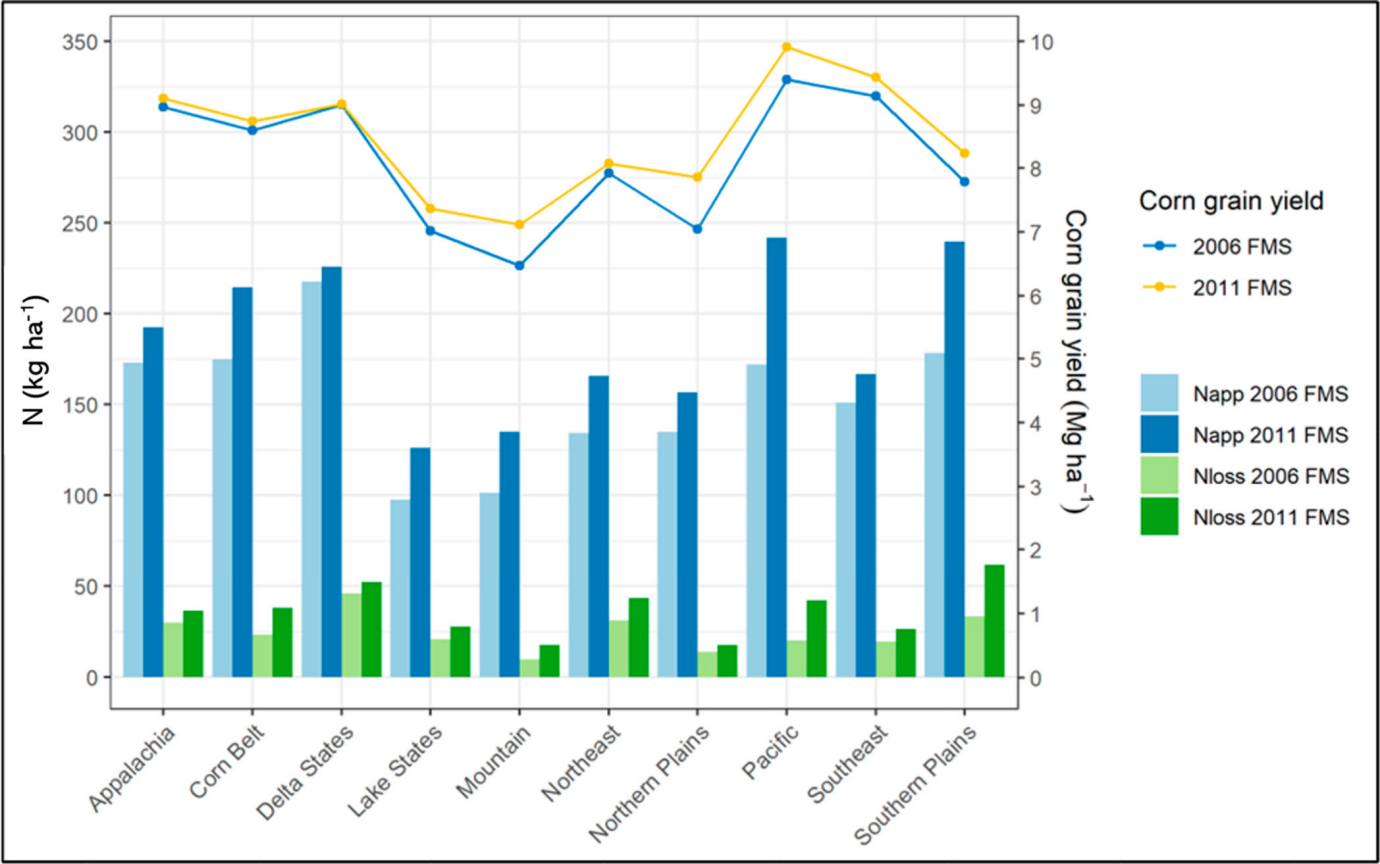
Comparisons of average annual N applications (fertilizer and manure), corn grain yields, and N losses from all loss pathways simulated for the 2006 and 2011 FMSs for each of the 10 production regions.

Across the production regions (Figure S1 in the Supplemental Materials), four regions (Mountain, Northeast, Lake States, and Northern Plains) in the 2006 FMS, accounting for about 38% of the corn grain areas—and two regions (Mountain and Lake States) in the 2011 FMS, accounting for about 25% of the corn grain areas—had the amount of N applied (N_app_) below 150 kg N ha^−1^ yr^−1^ (Table 1 and Figure 6). One region (Delta States) in the 2006 FMS, accounting for about 18% of the corn grain areas—and four regions (Pacific, Corn Belt, Delta States and Southern Plains) in the 2011 FMS, accounting for more than 50% of the corn grain areas—had the amount of N applied (N_app_) above 200 kg N ha^−1^ yr^−1^ (Table 1 and Figure 6).

**Figure 7.**
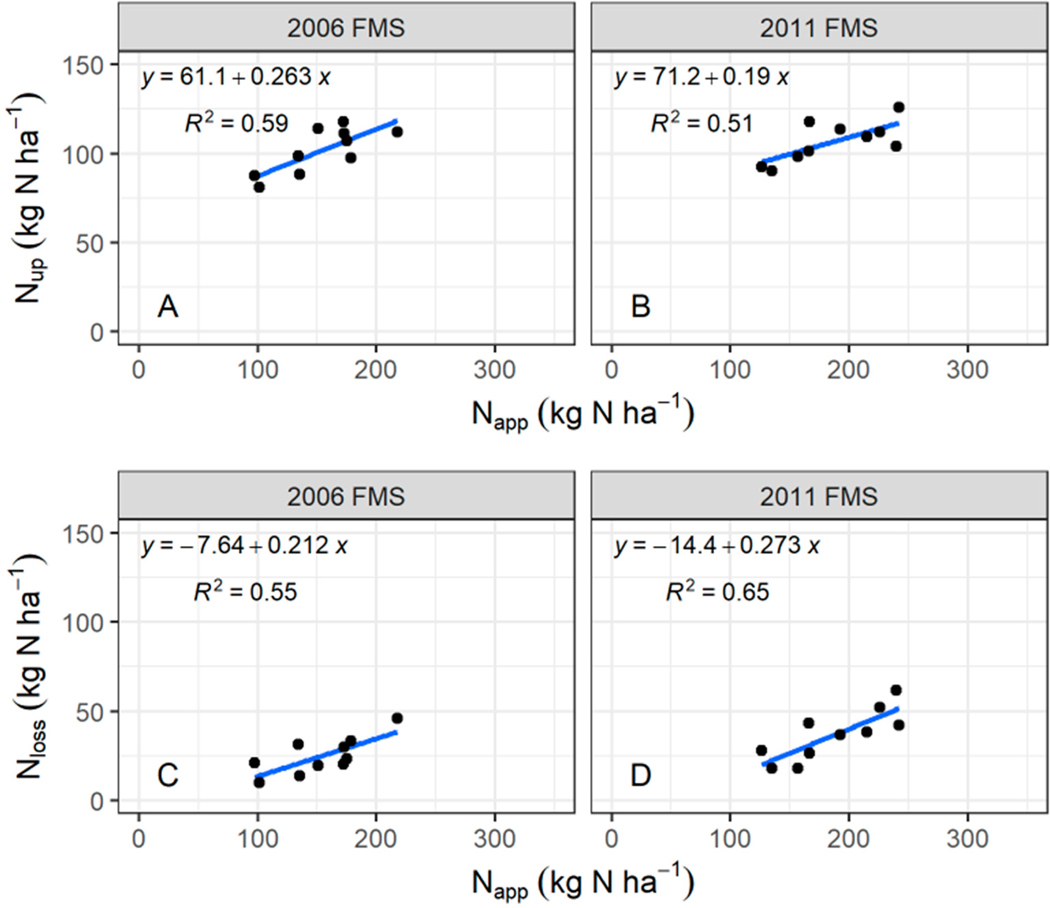
Area-weighted regional average annual N application vs. crop N uptake (**A**,**B**), N application vs. N losses (**C**,**D**).

Across the production regions, although corn grain yields from both FMSs followed the trend of N_app_ in general (Figure 6), a better correlation of N_up_ with N_app_ in the 2006 FMS (R^2^ = 0.60, Figure 7A) than in the 2011 FMS (R^2^ = 0.51, Figure 7B) was achieved, indicating that applied N was better utilized by crop in the 2006 FMS. The economic returns were minimal between the two FMSs because there was not much difference in corn grain yields. The NUEs were lower, with a higher N_app_ in the 2011 FMS than in the 2006 FMS (Table 2). The NUE was defined as the ratio of the N used by plant to the N applied. Corn grain yield reflected N used by plant in this study. The relationship between N_loss_ and N_app_ was stronger in the 2011 FMS (R^2^ = 0.65, Figure 7D) than that in the 2006 FMS (R^2^ = 0.55, Figure 7C), indicating that higher application is associated with higher N losses [15,42]. The increased amount of N_app_ not utilized by crops increased the potential of N loss to the environment through volatilization, denitrification, and leaching [43,44]. Liu et al. [42] summarized responses of corn grain yield to the N application rates for the Midwest and concluded that corn grain yield responded well to increased N application until the N application rate reached about 150 kg N ha^−1^ yr^−1^. Corn grain yield may still increase after N application rate of 150 kg N ha^−1^ yr^−1^, but at a slower pace. However, the responses of N losses to the N application rates were opposite to the responses of corn grain yield to the N application rates. In general, after a certain N application rate, higher N application rates resulted in higher N losses, but not much higher corn grain yields.

Increased N fluxes due to agricultural nonpoint source pollution from the Mississippi River Basin have been linked to increased occurrences of seasonal hypoxia in the northern Gulf of Mexico [45]. Boosting agricultural production such as fertilizer application can lead to environmental problems. Finding sustainable solutions in maintaining agricultural productivity while minimizing their adverse impacts on the environment is critical. Ideally, applied N would be removed with the harvested crop. Nitrogen mining is generally undesirable in the long term. Additionally, NUE > 1 can be achieved if the sum of the added N and the N available in the soil is less than the plant demand. However, this can be unsustainable for long-term crop production [34]. On the other hand, N levels can build up in the soil over time if N_up_ is lower than N_app_, which may be susceptible to runoff and leaching losses. The model simulated higher soil N gains in the 2011 FMS with more manure application, which is consistent with the assessment performed by the Conservation Effects Assessment Project (CEAP) cropland report [39].

## 4. Conclusions and Recommendations

In this study, the integrated meteorology and air quality WRF/CMAQ provided 12 km gridded daily weather input and atmospheric N deposition to EPIC to capture the effects of changing N inputs and weather on crop yield and N processes. The IMMMS 1.0 is responsive to different FMSs. Nitrogen application from inorganic sources (N_fer_) aggregated by state compared reasonably well with reported values. N_fer_ was slightly lower (about 1%) in the 2011 FMS than that in the 2006 FMS. However, N application from manure (N_man_) in the 2011 FMS was much higher than that in the 2006 FMS (41 kg N ha^−1^ yr^−1^ vs. 7 kg N ha^−1^ yr^−1^) (Table 1), which resulted in an overall 21.2% higher N application (N_app_) in the 2011 FMS than in the 2006 FMS (Table 1). Although applications of N-based fertilizers and manure nutrients were essential to maintain high crop yields, higher N_app_ in the 2011 FMS only led to slightly higher crop uptake and corn grain yield as well as a much higher total N loss relative to the 2006 FMS from this analysis. In addition, both scenarios captured the historical corn grain yield reported by NASS well. Furthermore, corn grain NUE values were relatively lower in the 2011 FMS, indicating that the economic returns were minimal at higher levels of N application in the 2011 FMS, which is consistent with the findings in the literature. The IMMMS 1.0 can evaluate the relative relationship of N application, crop yield, and N losses.

Over the CONUS, N loss through denitrification accounted for about 25% and 33% of total N loss in the two FMSs, respectively; N lost through volatilization accounted for about 29% (in the 2006 FMS) and 28% (in the 2011 FMS) of total N loss. The largest percentage of total N loss was through water and sediment, which accounted for about 46% and 39% of the total N loss in the 2006 and 2011 FMSs, respectively.

Future improvements include the refinement of crop management operations and crop rotations, refinement of soil physical and chemical properties, and examination of their influences on model predictions of crop yield, water, N, NUE, and C content in soil. In addition, future research needs to evaluate responses of N loading to the large river basins (such as the Mississippi River Basin) to different FMSs as well as to different climate scenarios so that we can gain better understanding of N processes and N loading changes, which would be very useful for the Hypoxia Task Force as they prepare for future responses to the hypoxia zone of the Gulf of America. With further improvements and evaluations, the IMMMS 1.0 can be better utilized to improve the understanding of N fertilization, agricultural production, weather, and their impacts on hydrology, water quality, and air quality at large river basin and/or national scales.

## Supplementary Material

Supplement1

**Supplementary Materials:** The following supporting information can be downloaded at: https://www.mdpi.com/article/10.3390/agriculture15101017/s1, references [46–55] are cited in Supplementary Materials.

## Figures and Tables

**Table 1.** Average annual N input from the 2006 and 2011 FMS at the 10 agricultural production regions over the simulation period from 2003 to 2017.

Region	Simulated Corn Area		N Applied (kg ha^−1^)						Percent Change Between FMSs (%)	
			2006 FMS			2011 FMS				
	(ha)	(%)	N_fer_	N_man_	N_app_	N_fer_	N_man_	N_app_	∆N_fer_	∆N_app_
Pacific	137,435	0.4	163.3	8.8	172.1	127.5	114.4	241.9	−21.9	40.6
Corn Belt	15,048,093	48.0	168.7	6.3	175.0	174.7	40.0	214.7	3.6	22.7
Mountain	470,802	1.5	96.5	4.8	101.3	69.3	65.6	134.9	−28.2	33.2
Northeast	652,780	2.1	120.1	14.3	134.4	89.4	76.5	165.9	−25.5	23.4
Southeast	411,157	1.3	141.8	9.3	151.1	118.2	48.6	166.8	−16.7	10.4
Appalachia	1,094,494	3.5	171.1	1.9	173.0	138.3	54.2	192.5	−19.2	11.3
Lake States	5,117,713	16.3	92.2	5.4	97.6	66.5	59.7	126.2	−27.9	29.4
Delta States	934,136	3.0	211.5	6.1	217.6	225.8	0.0	225.8	6.8	3.8
Northern Plains	6,763,499	21.6	126.1	9.0	135.1	139.9	16.9	156.8	11.0	16.1
Southern Plains	695,400	2.2	168.1	10.3	178.4	133.4	106.3	239.7	−20.7	34.3
All regions	31,325,510	100.0	145.9	6.8	152.7	144.5	40.6	185.1	−0.9	21.2

**Table 2.** Area-weighted average annual corn grain yield and plant nutrient uptake by region from 2006 and 2011 FMS simulations.

Region	2006 FMS			2011 FMS		
	Corn Grain Yield (Mg ha^−1^)	N_up_ (kg ha^−1^)	NUE	Corn Grain Yield (Mg ha^−1^)	N_up_ (Kg ha^−1^)	NUE
Pacific	9.40	117.8	0.68	9.91	126.1	0.52
Corn Belt	8.60	107.2	0.61	8.75	109.5	0.51
Mountain	6.47	81.2	0.80	7.12	90.4	0.67
Northeast	7.93	98.8	0.74	8.08	101.5	0.61
Southeast	9.14	113.9	0.75	9.44	117.9	0.71
Appalachia	8.97	111.5	0.64	9.11	113.7	0.59
Lake States	7.02	87.7	0.90	7.37	92.7	0.73
Delta States	9.01	112.0	0.51	9.02	112.2	0.50
Northern Plains	7.05	88.3	0.65	7.87	98.3	0.63
Southern Plains	7.79	97.4	0.55	8.24	104.0	0.43
All regions	7.98	99.6	0.65	8.32	104.2	0.56

**Table 3.** Average annual N loss estimates by region from corn grain area in the 2006 and 2011 FMSs.

Region	2006 FMS (kg ha^−1^)					2011 FMS (kg ha^−1^)				

	N_vol_	N_den_	N_wat_	N_sed_	N_loss_	N_vol_	N_den_	N_wat_	N_sed_	N_loss_

Pacific	9.2	8.7	2.0	0.2	20.1	14.6	23.5	4.2	0.1	42.3
Corn Belt	6.7	4.6	6.6	5.5	23.3	11.0	11.1	10.4	5.8	38.3
Mountain	5.9	1.7	1.6	0.7	9.9	6.9	7.5	2.5	0.8	17.8
Northeast	4.4	9.4	9.2	8.3	31.4	6.7	14.1	12.3	10.4	43.5
Southeast	4.4	8.3	4.6	2.3	19.6	7.0	12.6	4.4	2.4	26.4
Appalachia	5.9	10.0	9.9	4.3	30.0	8.6	12.6	10.3	5.3	36.7
Lake States	5.7	3.8	6.9	4.6	21.0	7.9	6.8	9.0	4.2	27.8
Delta States	5.3	25.0	13.2	2.5	46.1	6.6	27.6	15.6	2.6	52.3
Northern Plains	6.1	3.5	1.4	2.7	13.8	7.5	6.4	1.7	2.1	17.8
Southern Plains	6.7	18.4	4.5	3.8	33.4	10.7	41.7	4.7	4.6	61.8
All regions	6.3	5.4	5.7	4.5	21.9	9.3	10.7	8.1	4.6	32.7
Change(%)	47.6	98.1	42.1	2.2	49.3					

## Data Availability

Data are available upon request.

## References

[1] Ladha JK; Tirol-Padre A; Reddy CK; Cassman KG; Verma S; Powlson DS; van Kessel C; Richter DD; Chakraborty D; Pathak H Global nitrogen budgets in cereals: A 50-year assessment for maize, rice, and wheat production systems. Sci. Report 2016, 6, 19355. 10.1038/srep19355 PMC4726071 26778035

[2] Fowler D; Coyle M; Skiba U; Sutton MA; Cape JN; Reis S; Sheppard LJ; Jenkins A; Grizzetti B; Galloway JN; The global nitrogen cycle in the twenty-first century. Philos. Trans. R. Soc. Lond. B Biol. Sci 2013, 368, 1621. 10.1098/rstb.2013.0164 PMC3682748 23713126

[3] Galloway JN; Dentener FJ; Capone DG; Boyer EW; Howarth RW; Seitzinger SP; Asner GP; Cleveland CC; Green PA; Holland EA; Nitrogen cycles: Past, present, and future. Biogeochemistry 2004, 70, 153–226.

[4] Sutton MA; Bleeker A; Howard C; Bekunda M; Grizzetti B; Vries W.d.; Van Grinsven H; Abrol Y; Adhya T; Billen G Our Nutrient World: The Challenge to Produce More Food and Energy with Less Pollution; NERC/Centre for Ecology & Hydrology: Edinburgh, UK, 2013; 114p.

[5] Almasri MN; Kaluarachchi JJ Assessment and management of long-term nitrate pollution of ground water in agriculture dominated watersheds. J. Hydrol 2004, 295, 225–245.

[6] Bouwman AF; Van Drecht G; Knoop JM; Beusen AHW; Meinardi CR Exploring changes in river nitrogen export to the world’s oceans. Glob. Biogeochem. Cycles 2005, 19, GB1002.

[7] Goolsby DA; Battaglin WA; Aulenbach BT; Hooper RP Nitrogen input to the Gulf of Mexico. J. Environ. Qual 2001, 30, 329–336. 11285892 10.2134/jeq2001.302329x

[8] Rockstrom J; Steffen W; Noone K; Persson A; Chapin FS; Lambin EF; Lenton TM; Scheffer M; Folke C; Schellnhuber HJ; A safe operating space for humanity. Nature 2009, 461, 472–475. 19779433 10.1038/461472a

[9] Vitousek PM; Naylor R; Crews T; David MB; Drinkwater LE; Holland E; Johnes PJ; Katzenberger J; Martinelli LA; Matson PA; Nutrient imbalances in agricultural development. Science 2009, 324, 1519–1520. 19541981 10.1126/science.1170261

[10] Lu C; Tian H Net greenhouse gas balance in response to nitrogen enrichment: Perspectives from a coupled biogeochemical model. Glob. Change Biol 2013, 19, 571–588. 10.1111/gcb.12049 23504794

[11] Zhang X; Davidson EA; Mauzerall DL; Searchinger TD; Dumas P; Shen Y Managing nitrogen for sustainable development. Nature 2015, 528, 51–59. 26595273 10.1038/nature15743

[12] Lu C; Tian H Global nitrogen and phosphorus fertilizer use for agriculture production in the past half century: Shifted hot spots and nutrient imbalance. Earth Syst. Sci. Data 2017, 9, 181–192.

[13] Searchinger T; Waite R; Hanson C; Ranganathan J; Matthews E Creating a Sustainable Food Future: A Menu of Solutions to Sustainably Feed More than 9 Billion People by 2050. World Resources Report. Interim Findings; World Resources Institute: Washington, DC, USA, 2019.

[14] Davidson E; Galloway J; Millar N; Leach A N-related greenhouse gases in North America: Innovations for a sustainable future. Curr. Opin. Environ. Sust 2014, 9–10, 1–8.

[15] Liu WH; Yang J; Liu LB; Azevedo X; Wang Z; Xu KC; Abbaspour R; Schulin R Global assessment of nitrogen losses and trade-offs with yields from major crop cultivations. Sci. Total Environ 2016, 572, 526–537. 27552131 10.1016/j.scitotenv.2016.08.093

[16] USEPA SAB (United States Environmental Protection Agency Science Advisory Board). Reactive Nitrogen in the United States: An Analysis of Inputs, Flows, Consequences, and Management Options; USEPA SAB (United States Environmental Protection Agency Science Advisory Board): Washington, DC, USA, 2013; Volume EPA-SAB-11–013, pp. 1–218.

[17] Sutton MA; Howard CM; Erisman JW The European Nitrogen Assessment: Sources, Effects, and Policy Perspectives; Cambridge University Press: Cambridge, UK, 2011.

[18] Yuan Y; Wang R; Cooter E; Ran L; Daggupati P; Yang D; Srinivasan R; Jalowska A Integrating multimedia models to assess nitrogen losses from the Mississippi River basin to the Gulf of Mexico. Biogeosciences 2018, 15, 7059–7076. 31320910 10.5194/bg-15-7059-2018 PMC6638569

[19] Cooter EJ; Bash JO; Benson V; Ran L Linking agricultural crop management and air quality models for regional to national-scale nitrogen assessments. Biogeosciences 2012, 9, 4023–4035.

[20] Pleim JE; Ran L; Appel W; Shephard MW; Cady-Pereira K New bidirectional ammonia flux model in an air quality model coupled with an agricultural model. Adv. Model. Earth Syst 2019, 11, 2934–2957. 10.1029/2019MS001728 PMC7970535 33747353

[21] Ran L; Yuan Y; Cooter E; Benson V; Yang D; Pleim J An integrated agriculture, atmosphere, and hydrology modeling system for ecosystem assessments. Adv. Model. Earth Syst 2019, 11, 4645–4668. 10.1029/2019MS001708 PMC8193828 34122728

[22] Williams JR The EPIC model. In Computer Models of Watershed Hydrology; Singh VP, Ed.; Water Resources Publications: Highlands Ranch, CO, USA, 1995; pp. 909–1000.

[23] Wang X; Kemanain A; Williams J Special features of the EPIC and APEX modeling package and procedures for parameterization, calibration, validation, and applications. In Methods of Introducing System Models into Agricultural Research. Advances in Agricultural Systems Modeling 2; Ahuja L, Ma L, Eds.; ASA, CSSA, SSSA: Madison, WI, USA, 2011; pp. 177–208.

[24] Williams JR The Erosion-Productivity Impact Calculator (EPIC) Model: A Case History. Phil. Trans. R. Soc. Lond. B 1990, 329, 421–428.

[25] Wang X; Williams JR; Gassman PW; Baffaut C; Izaurralde RC; Jeong J; Kiniry JR EPIC and APEX: Model Use, calibration, and validation. Trans. ASABE 2012, 55, 1447–1462.

[26] Arnold JG; Moriasi DN; Gassman PW; Abbaspour KC; White MJ; Srinivasan R; Santhi C; Harmel RD; Griensven AV; Liew MW; SWAT: Model use, calibration, and validation. Trans. ASABE 2012, 55, 1491–1508.

[27] Gassman PW; Reyes MR; Green CH; Arnold JG The soil and water assessment tool: Historical development, applications, and future research directions. Trans. ASABE 2007, 50, 1211–1250.

[28] Neitsch SL; Arnold JG; Kiniry FR; Williams JR Soil and Water Assessment Tool (Version 2009): Theoretical Documentation; USDA-ARS Grassland, Soil and Water Research Laboratory and Blackland Research Center: Temple, TX, USA, 2011.

[29] Chaplot V; Saleh A; Jaynes DB; Arnold J Predicting water, sediment and NO3-N loads under scenarios of land-use and management practices in a flat watershed. Water Air Soil Pollut. 2004, 154, 271–293.

[30] Johnson T; Butcher J; Deb D; Faizullabhoy M; Hummel P; Kittle J; Witt J Modeling Streamflow and Water Quality Sensitivity to Climate Change and Urban Development in 20 US Watersheds. J. Am. Water Resour. Assoc 2015, 51, 1321–1341. 36203498 10.1111/1752-1688.12308 PMC9534033

[31] Santhi C; Srinivasan R; Arnold JG; Williams JR A modeling approach to evaluate the impacts of water quality management plans implemented in a watershed in Texas. Environ. Model. Softw 2006, 21, 1141–1157.

[32] Vaché KB; Eilers JM; Santelmann MV Water quality modeling of alternative agricultural scenarios in the US corn belt. J. Am. Water Resour. Assoc 2002, 38, 773–787.

[33] US Department of Agriculture, National Agricultural Statistics Service; National Agricultural Statistics Service. Corn Yield by Year, 2022. Available online: https://www.nass.usda.gov/Charts_and_Maps/Field_Crops/cornyld.php (accessed on 19 April 2025).

[34] Fixen PE; Brentrup F; Bruulsema T; Garcia F; Norton R; Zingore S Nutrient/Fertilizer Use Efficiency: Measurement, Current Situation and Trends. In Managing Water and Fertilizer for Sustainable Agricultural Intensification; Drechsel P, Heffer P, Magen H, Mikkelsen R, Wichelns D, Eds.; IFA: Paris, France, 2015; ISBN 979–10-92366–02-0.

[35] Snyder CS; Bruulsema TW Nutrient Use Efficiency and Effectiveness in North America: Indices of Agronomic and Environmental benefit; International Plant Nutrition Institute: Norcross, GA, USA, 2007.

[36] Richards RP; Baker DB; Eckert DJ Trends in agriculture in the LEASEQ watersheds, 1975–1995. J. Environ. Qual 2002, 31, 17–24. 11837420 10.2134/jeq2002.1700

[37] Forster DL; Rausch JN Evaluating agricultural nonpoint source pollution programs in two Lake Erie tributaries. J. Environ. Qual 2002, 31, 24–31. 11837427 10.2134/jeq2002.2400

[38] Blevins RL; Frye WW Conservation tillage: An ecological approach to soil management. Adv. Agron 1993, 51, 33–78.

[39] Kern JS; Johnson MG Conservation tillage impacts on national soil and atmospheric carbon levels. Soil Sci. Soc. Am. J 1993, 57, 200–210.

[40] US Department of Agriculture; Natural Resources Conservation Service. Effects of Conservation Practices on Nitrogen Loss from Farm Fields: A National Assessment Based on the 2003–06 CEAP Survey and APEX Modeling Databases; 2017; 129p. Available online: https://www.nrcs.usda.gov/publications/ceap-crop-2017-nitrogen-loss.pdf (accessed on 19 April 2025).

[41] Robertson GP Nitrogen Use Efficiency in Row-Crop Agriculture: Crop Nitrogen Use and Soil Nitrogen Loss. In Ecology in Agriculture; Jackson LE, Ed.; Academic Press: New York, NY, USA, 1997; pp. 347–365.

[42] Liu W; Yang H; Folberth C; Muller C; Ciais P; Abbaspour KC; Schulin R Achieving high crop yields with low nitrogen emissions in global agricultural input intensification. Environ. Sci. Technol 2018, 52, 13782–13791. 30412669 10.1021/acs.est.8b03610

[43] Cassman K; Dobermann A; Walters DT Agroecosystems, nitrogen-use efficiency, and nitrogen management. Ambio 2002, 31, 132–140. 12078002 10.1579/0044-7447-31.2.132

[44] Tilman D; Cassman KG; Matson PA; Naylor R; Polasky S Agricultural sustainability and intensive production practices. Nature 2002, 418, 671–677. 12167873 10.1038/nature01014

[45] United States Environmental Protection Agency. Mississippi River Gulf of Mexico Watershed Nutrient Task Force: New Goal Framework 2014; Office of Wetlands, Oceans, and Watersheds: Washington, DC, USA, 2014.

[46] Cohan DS; Boylan JW; Marmur A; Khan MN An integrated framework for multipollutant air quality management and its application in Georgia. Environ. Manag 2007, 40, 545–554. 10.1007/s00267-006-0228-4 17638048

[47] Williams JR; Arnold JG Water quality models for watershed management. In Water-Quality Hydrology; Springer Nature: Berlin/Heidelberg, Germany, 1996; pp. 217–241.

[48] Skamarock WC; Klemp JB; Dudhia J; Gill DO; Barker DM; Wang W; Powers JG A Description of the Advanced Research WRF, Version 3; NCAR Tech Note, 1035 NCAR/TN 475+STR; National Center for Atmospheric Research: Boulder, CO, USA, 2008.

[49] Bash JO; Cooter E; Dennis RL; Walker JT; Pleim JE Evaluation of a regional air-quality model with bidirectional NH3 exchange coupled to an agroecosystem model. Biogeosciences 2013, 10, 1635–1645.

[50] Pleim JE; Bash JO; Walker JT; Cooter E Development and evaluation of an ammonia bidirectional flux parameterization for air quality models. J. Geophys. Res 2013, 118, 3794–3806.

[51] Byun DW; Schere KL Review of the governing equations, computational algorithms, and other components of the models-3 community multiscale air quality (CMAQ) modeling system. Appl. Mech. Rev 2006, 59, 51–77.

[52] Appel KW; Napelenok SL; Foley KM; Pye HOT; Hogrefe C; Luecken DJ; Bash JO; Roselle SJ; Pleim JE; Foroutan H; Description and evaluation of the Community Multiscale Air Quality (CMAQ) modeling system version 5.1. Geosci. Model Dev 2017, 10, 1703–1732. 30147852 10.5194/gmd-10-1703-2017 PMC6104654

[53] Fu X; Wang SX; Ran LM; Pleim JE; Cooter E; Bash JO; Benson V; Hao JM Estimating NH3 emissions from agricultural fertilizer application in China using the bi-directional CMAQ model coupled to an agro-ecosystem model. Atmos. Chem. Phys 2015, 15, 6637–6649.

[54] Vinten AJA; Smith KA Nitrogen cycling in agricultural soils. In Nitrate: Processes, Patterns and Management 1993; Burt TP, Heathwaite AL, Trudgill ST, Eds.; Wiley: Chichester, UK, 1993; pp. 39–73.

[55] Goebes MD; Strader R; Davidson C An ammonia emission inventory for fertilizer application in the United States. Atmos. Environ 2003, 37, 2539–2550.

