# Supplementary material for: An Integrated Multi-Media Modeling System for Regional- to National-Scale Nitrogen and Crop Productivity Assessments
Source: Agriculture (Basel). Author manuscript; Available in PMC 2025 Aug 11. (PMC12338305; doi:10.3390/agriculture15101017)
Supplement: Supplement1 [file NIHMS2091388-supplement-Supplement1.pdf]

An Integrated Multi-Media Modeling System for Regional to National-scale Nitrogen and Crop Productivity Assessments

Yongping Yuan [1*], Susan Wang [2], Verel. Benson [3], and Limei Ran [4]

1. Hydrologist at US Environmental Protection Agency-Office of Research and Development, Research Triangle Park, North Carolina 27711, USA; yuan.yongping@epa.gov

2. Carbon Modelling Lead at AGORO; susan.wang@agorocarbon.com

3. Retiree at Mizzou University of Missouri, Columbia, Missouri, USA; BensonV@missouri.edu.

4. Senior Scientist at US Department of Agriculture Natural Resources Conservation Service Resource Inventory and Assessment Division; Limei.Ran@usda.gov.

[*]Corresponding author: Yongping Yuan, yuan.yongping@epa.gov, 109 T.W. Alexander Dr. RTP, NC 27711

Supplemental Materials

List of abbreviations

Acronym	Definition
CAFO	Confined Animal Facility Operation
C	Carbon
CMAQ	Community Multiscale Air Quality
COA	Census of Agriculture
CONUS	Contiguous United States
EPIC	Environmental Policy Integrated Climate
FEST-C	Fertilizer Emission Scenario Tool for CMAQ
FMS	Fertilizer management scenario
IMMMS	Integrated multi-media modelling system
N	Nitrogen
NASS	National Agricultural Statistics Service
NLCD	National Land Cover Database
NUE	N use efficiency
SWAT	Soil and Water Assessment Tool
USDA	United States Department of Agriculture
USEPA	United States Environmental Protection Agency
WRF	Weather Research and Forecasting

1. Introduction

1.1 IMMMS Development History and Applications

Elevated levels of nitrogen (N) in surface water can originate from a variety of sources, including discharges from sewage treatment plants, deposition of atmospheric N, and stormwater runoff from both urban and agricultural fields. Agricultural runoff was thought to be the major sources of nitrogen entering the Gulf of America (GOA) via the Mississippi River, leading to the largest hypoxic zone (dead zone) in the U.S. Various modeling studies, conducted to improve our understanding of factors and sources contributing to increased N export from the Mississippi River Basin (MRB), have been focused on interactions of land and water. However, it is not clear how atmospheric N deposition contributes to the total N load and its impact on rivers, lakes and estuaries. Furthermore, climate is changing: temperatures are rising, snow and rainfall patterns are shifting, and more extreme climate events such as heavy rainstorms and record high temperatures are happening. Considering the expected changes in climate during N assessment is also critical, because future climate scenarios may impact streamflow generation, and thus, N loads from the watershed. Finally, due to the complex N cycle and its dynamics from the atmosphere to the biosphere, through dry deposition of gaseous N species and wet deposition of dissolved N species in precipitation, there is a need for integrated, multimedia and transdisciplinary approaches to evaluate N sources, fate and transport comprehensively.

The USEPA has developed the Community Multiscale Air Quality (CMAQ, https://www.epa.gov/cmaq) Modelling System, which is a sophisticated three-dimensional Eulerian grid chemical transport model for studying air pollution from local to hemispheric scales; while the Weather Research and Forecasting (WRF) is a community next-generation mesoscale numerical weather prediction system designed for atmospheric research and forecasting applications by the US National Center for Atmospheric Research (https://www.mmm.ucar.edu/weather-research-and-forecasting-model). The integrated meteorology and air quality modeling system WRF/CMAQ is an important decision support tool that has been used to help understand the chemical and physical processes for research and policy-making to mitigate harmful effects of air pollution on human health and the environment around the world [46].

However, during air quality simulations, it is often a challenge to accurately estimate NH_3 emissions from agricultural land because N fertilization varies spatially and temporally by production types (e.g. corn vs. soybean) and locations (e.g. different soil and weather). Therefore, the USEPA developed the Fertilizer Emission Scenario Tool for CMAQ (FEST-C) system [18], an advanced user interface, to integrate the Environmental Policy Integrated Climate (EPIC) model [22; 24; 47], a field-scale agricultural biogeochemical model, with WRF model [48] and CMAQ; and WRF/CMAQ simulates mesoscale meteorology and air quality (Fig. 1). The FEST-C system (EPIC/WRF/CMAQ) simulates daily fertilizer application to agricultural lands for bi-directional ammonia (NH_3) modelling [49-50] in the CMAQ model [51-52] and is useful for assessing impacts of agricultural fertilization and management practices not only on air quality (NH_3) [53] and climate (nitrous oxide (N_2O)) [18], but also on crop yield, soil erosion, and hydro-ecosystems. The FEST-C system (EPIC/WRF/CMAQ) consists of field-scale models, thus the Soil and Water Assessment Tool (SWAT), a widely applied watershed hydrology and water quality model [26-28], was integrated with the FEST-C system (EPIC/WRF/CMAQ) to

address watershed processes and/or watershed hydrology and water quality for a full multimedia assessment on water quality [19]. Subsequently, this modeling system was named as integrated multi-media modelling system (IMMMS). Table S1 summarized past studies performed on this modeling system.

Table S1: Summary of past applications of the modeling system.

Authors	Title	Models	Domain	Simulation period	FMS	Focus of the study
Cooter et al., 2012 [18]	Linking agricultural crop management and air quality models for regional to national-scale nitrogen assessments	FEST-C EPIC	12-km CONUS*<br>Corn grain in the Southeast production region.	2002	2006	Description of Integration of EPIC with WRF/CMAQ, EPIC inputs development, and evaluation of fertilizer management scenario (2006 FMS) through comparing with other data sources (e. g. 2001 Inorganic N use by Ruddy et al. (2006).
Yuan et al., 2018 [19]	Integrating multimedia models to assess nitrogen losses from the Mississippi River basin to the Gulf of Mexico	IMMMS-SWAT	Mississippi River Basin (MRB)	2002-2010	2006	Description of Integration of SWAT with FEST-C EPIC, evaluation of dissolved nitrogen simulated by SWAT (SWAT was run both with and without integration with EPIC) through comparing with USGS observations near the basin outlet.
Pleim et al., 2019 [20]	New Bidirectional Ammonia Flux Model in an Air Quality Model Coupled With an Agricultural Model	IMMMS<br>CMAQ	12-km CONUS*	2015 to 2016	2006	Evaluation of ammonia concentrations of CMAQ (CMAQ was run both with and without the new EPIC bidirectional NH_3 flux model) through

						comparing with observations from ammonia monitoring network
Ran et al., 2019 [21]	An Integrated Agriculture, Atmosphere, and Hydrology Modeling System for Ecosystem Assessments	IMMMS	12-km CONUS*<br>Agricultural fields	2010, 2011 and 2012	2006	Description of IMMMS, evaluation of water budget and irrigation demand, nitrogen budget with fertilization, Ambient gas-phase NH_3 concentration, and NH_4 wet deposition
Yuan et al., 2025	An Integrated Multi-Media Modeling System for Regional to National-scale Nitrogen and Crop Productivity Assessments	FEST-C EPIC	12-km CONUS*	2003-2017	2006<br>2011	Evaluation of FMS 2011 by comparing simulation results with FMS 2006, evaluation of corn grain yield through comparison with USDA NASS reported corn grain yield.

*CONUS: contiguous United States

2. Methods and Procedures

2.1 Nitrogen Processes Simulated in EPIC

EPIC has been designed to provide a full biogeochemical characterization of agricultural systems since its original development. Briefly, it simulates the complete N cycle: atmospheric N inputs; fertilizer/manure N applications; crop N uptake; nitrification (transformation of the NH_4^+ pool to NO_3^-); denitrification (conversion of NO_3^- to produce N_2 and N_2O); ammonia volatilization (gaseous loss of NH_3 that occurs when NH_4^+ is surface applied); decomposition; mineralization and immobilization; organic N transport on sediment; and nitrate-N losses in leaching, surface runoff, lateral subsurface flow and tile flow. Mineralization is the process that breaks down organic N compounds in the soil to release NH_4^+, with concurrent release of C as CO_2 in most cases [55]; the reverse process is immobilization by which NH_4^+ pool to NO_3^- are microbially transformed into organic forms. Decomposition and mineralization of fresh organic N are controlled by a decay rate constant. Denitrification occurs only when soil moisture content is above field capacity. The fertilizer N is considered to dissolve immediately and contribute to the mineral N pool. Plant uptake of N is controlled by plant demand, but limited by soil supply of the N. Organic N in each soil layer is partitioned into fresh and stable pools. The organic N loss is estimated using sediment yield, organic N on the soil surface layer, and an enrichment ratio; the soluble N loss is estimated by considering soluble N concentration changes in soil layers [29]. EPIC was modified to accept time series of wet and dry atmospheric deposition of oxidized and reduced N species from WRF/CMAQ through the FEST-C system [18; 21]. EPIC also includes a heat unit-driven, above and below ground plant growth model, soil hydrology and soil heat budgets for multiple soil layers of variable thickness. Simulation output frequency is user-specified, ranging from daily to annual summaries of biogeochemical process rates, nutrient pools and management activity, as well as edge-of-field runoff, sediment, and nutrients.

2.2. Initial Model Inputs and Configuration

To facilitate the integration of EPIC with WRF/CMAQ and make the system more user friendly, EPIC required inputs regarding soils, crop area, crop management were developed and stored as common data for this modeling system. Details on development for those datasets are descript in [18]. Specifically, for crop and crop management, twenty-one crops are simulated (Table S2). The United States Geological Survey (USGS) National Land Cover Database (NLCD) was used to provide spatial details of land use and land cover (http://landcover.usgs.gov/uslandcover.php) for the Contiguous United States (48 states). Fractions of crop land in each simulation grid (12 by 12 km) were assigned based on a county-level spatial crop assignment which was obtained from USDA National Agricultural Statistics Service (NASS) Census of Agriculture (http://www.agcensus.usda.gov/Publications). It was assumed that each 12 km model grid contains multiple EPIC monoculture “fields”, but the location of each field within a grid is spatially indeterminate. Agricultural area in a grid is determined using the 30m 2001 NLCD data layer (classes 81 and 82), and the distribution of specific crops within these NLCD grid areas is determined using the USDA county crop statistics. Each 12 km grid is assigned to a county polygon and is assumed to mirror that county’s crop distribution. When a grid spans multiple county polygons, the NLCD-determined agricultural area is assigned proportionally to each county, and the appropriate county crop

distribution is applied to those area fractions. An EPIC field, then, is defined as the agricultural area assigned to a specific crop within a 12 km grid. There can be up to 42 “fields” (21 rainfed or irrigated crops, see Table S3) in a grid. Specific crop and soil combinations vary by 8-digit HUC, and crop-specific management such as fertilization varies on an agricultural production area. EPIC is then run for each crop scenario in each grid across the full model domain. These results are then area weighted to aggregate grid estimates of fertilizer inputs, which are then shared with the regional air quality model.

Table S2: Crops modeled within the IMMMS.

Crop Name	Rainfed Crop Code [a]	Irrigated Crop Code
Grass Hay	22	23
Alfalfa	24	25
Other grazed cropland and pasture	26	27
Barley	28	29
Edible dry beans	30	31
Corn for grain	32	33
Corn for silage	34	35
Cotton	36	37
Oats	38	39
Peanuts	40	41
Potatoes	42	43
Rice	44	45
Rye	46	47
Sorghum for grain	48	49
Sorghum for silage	50	51
Soybeans	52	53
Spring wheat	54	55
Winter wheat	56	57
Other crops	58	59
Canola	60	61
Edible dry peas	62	63

The crop nutrient interactions are complicated processes. One hand, limiting fertilization may limit crop growth which in turn reducing crop needs for fertilization; on the other hand,

fertilization boosts crop growth which in return increasing crop needs for fertilization. EPIC was initially run for a 25-yr spin-up period to allow nutrient pools and soil characteristics to adjust to the defined management environment and provide initial conditions for simulation of year-specific weather. The average annual plant demand N determined during the last 5-yr of this spin-up was used to guide fertilization scenario development which determines timing, amount and type of fertilizers applied for each crop. This is an iteration process that the total sum of use by crops should be limited by available fertilizers for crop use, which were the fertilizer sale data for 2006 FMS and fertilizer sale data and nutrient from manure for 2011. To make initial development simple and achievable, only commercial fertilizations were considered. Thus, only fertilizer sale data were used for crop use. Below described the development of 2011 fertilizer management scenario.

2.3 Development of 2011 fertilizer management scenario

For the 2011 FMS development, we followed the same principal. The databases and programs used to create the input for the FEST-C Modeling System use logic, physical relationships, weather characteristics, soil characteristics, survey data and expert judgement to estimate the amount, timing, and type of fertilizer applied to 21 rainfed and 21 irrigated crops at a 12 km-by-12 km grid level (Table S2). Actual fertilizer applied and timing varies by individual farmer because farmers are subject to illness, social events, and equipment failure that cannot be modeled or are not surveyed at that level of detail. However, there is a random effect of planting delays by crop and grid in the management generation program that introduces some variability. There is no attempt to vary the type of fertilizer by crop below a regional level. Farmers do vary their fertilizer types and rates as market prices and availability change; however, capturing such variation is very difficult and beyond the scope of this study. There is no consistent source of information to estimate this variability at a 12 km grid level.

2.3.1. Types of fertilizers

The types of fertilizers applied are based on fertilizer sales by state and manure from confined animal facility operation (CAFO). Again, only fertilizer sales by state were used for 2006 FMS. The state sales data are summarized for major fertilizer types by six-month period for each of USDA 10 agricultural production regions (Fig. S1). Most recent analyses selected a subset of fourteen commercial fertilizers and four manures from CAFO production for beef feedlots, dairy, confined swine and poultry production to summarize and allocate by crop for the 2011 fertilizer scenario (Table S3). These selected commercial fertilizers accounted for over 95% of major fertilizer sales. Nutrient contents of manure fertilizers are based on USDA Conservation Effectiveness Assessment Project (CEAP) estimates (Table S3).

1	Northeast	3	Southeast	5	Corn Belt	7	Northern Plains	9	Mountain
2	Appalachia	4	Lake States	6	Delta States	8	Southern Plains	10	Pacific

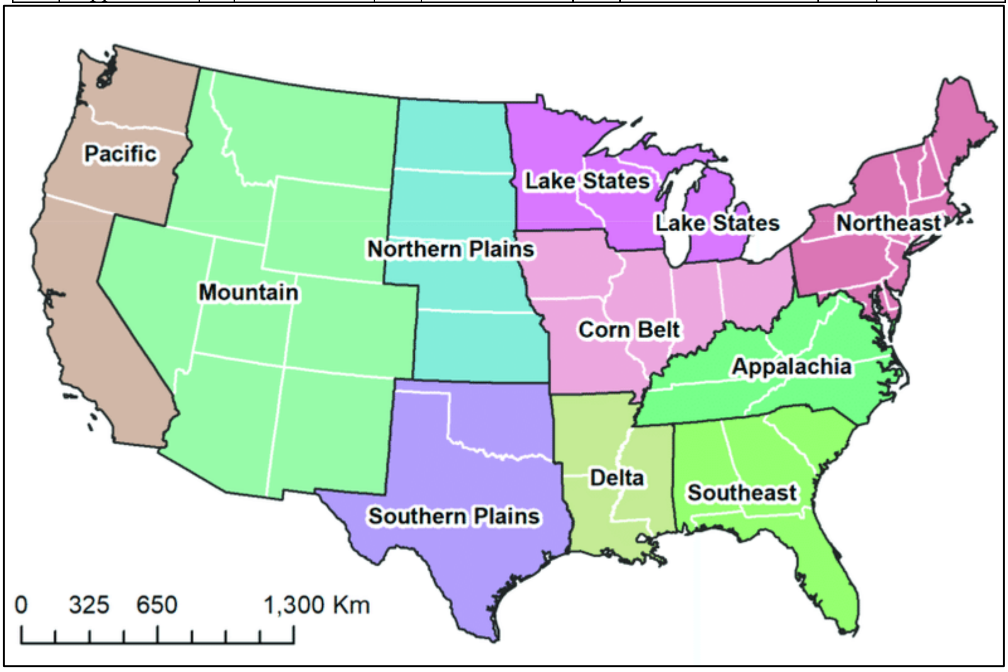

Figure S1: The USDA agricultural production regions.

Table S3. Commercial fertilizers and manures used in the 2011 fertilizer scenario.

Fertilizer		Fraction [a] (%)					
FertNumEPIC [b]	Description	FN: Mineral N	FP: Mineral P	FNO: Organic N	FPO: Organic P	FNH3 [c]: Ammonia N (FNH3/FN)	FOC: Organic C
22	Elemental N	1.0000	-	-	-	-	-
23	Elemental P	-	1.0000	-	-	-	-
25	Anhydrous Ammonia	0.8200	-	-	-	1.0000	-
27	Ammonium Nitrate	0.3400	-	-	-	0.5000	-
32	Ammonium Sulfate	0.2100	-	-	-	1.0000	-
45	Nitrogen Solution 28% (6.91%NO3, 6.91%NH3, and 14.23% Urea)	0.2800	-	-	-	0.7537	-
46	Nitrogen Solution 30%	0.3000	-	-	-	0.7538	-
47	Nitrogen Solution 32%	0.3200	-	-	-	0.7534	-
51	Urea	0.4600	-	-	-	1.0000	0.2000
58	Diammonium Phosphate	0.1800	0.2008	-	-	1.0000	-
59	Ammonium Polyphosphate	0.1500	0.2619	-	-	1.0000	-
64	Monoammonium Phosphate	0.1100	0.2269	-	-	1.0000	-
76	Liquid Ammonium Polyphosphate	0.1000	0.1484	-	-	1.0000	-
80	Superphosphate, Triple	-	0.2008	-	-	-	-

104	CEAP-104: Beef Solids 4 Barn, shed, or house 30% TS	0.0196	0.0133	0.0293	0.0089	0.7000	0.7896
118	CEAP-118: Dairy Solids 4 Barn, shed, or house 18% - 25% TS	0.0354	0.0199	0.0531	0.0066	0.5000	0.7522
129	CEAP-129: Swine solids all	0.0680	0.0289	0.0680	0.0192	0.9900	0.6935
136	CEAP-136: Poultry solids liter in house	0.0065	0.0117	0.0588	0.0078	0.9800	0.7779

[a]: These values are listed in the EPIC model input database file FERT2010.dat, which contains most common fertilizers and/or other nutrient materials used in agricultural management.
[b]: FertNumEPIC: linked with Tables 3 and 4 as column "FertNumEPIC".
[c]: The fraction of mineral nitrogen in the fertilizer that is in the ammonia (NH_3) form.

2.3.2. Fertilizer allocation: type, timing, and percentages of N and P needed for each crop

Sensible allocation of N and P were based on USDA data, fertilizer sales data, and recommendations from knowledgeable agricultural experts. The goal of the allocation process is to estimate the percentages of N and P needed for 21 rainfed and 21 irrigated crops (Table S2) in each of the 10 production regions that is supplied by each of 18 fertilizer sources where 14 are commercial fertilizers and 4 are manures as listed in Table S3. The total of the percentages is split by time of application into the following periods:

Code in database (see Tables S4 and S5: TimingID): Description

- 2- F-B4 plt&: Fall pre-plant and at plant
- 3- S with Fa: Spring after fall plant
- 4- S B4 plt: Spring pre-plant
- 5- S at plt: Spring at plant, and
- 6- S After p: Post plant application.

This level of data is not available from any database; however, cropping practice surveys provide some insight on application times by crop for some fertilizers. Agricultural census data provides estimates of crop production by state and region. This data is not always split into irrigated versus rainfed. The allocation methodology uses agricultural census estimates of N and P removal as an estimate of crop needs. Cropping practices surveys are used as indicators of timing of fertilization by the previously identified timing categories. Fertilizer sales by 6-month period also are indicators of availability of fertilizer by timing period. With this information in mind, the allocation table is filed out one crop at a time such that the total fertilizer sales by 6-month period and the N and P needs based on agricultural census production are balanced as well as possible. There is considerable subjectivity in this process due to lack of specific crop/fertilizer data; however, keep in mind that this is trying to estimate regional pollutant loads to air and water. This method does allocate N and P sources to crops within each region. In addition, we have created a system that can simulate alternative fertilizer use by crop, type of fertilizer and timing to estimate potential impacts of nutrient management on pollutant loading to air and water.

Manure is assumed to be allocated to silage crops and hay to meet P needs because these crops are often co-located with animal production. Corn grain is assumed to be the next crop to which manure might be applied. There are crops with limited or no information, such as hay, alfalfa, other grass, edible beans, peanuts, potatoes, other crop, canola, and peas. For these 100%

identified by grade N and 100% identified by grade P2O5 can be a default unless there have reason to allocate otherwise. This just makes sure that fertilizer is applied to meet crop removal with adjustments for nitrogen fixation with legumes where fixed N is assumed to be 10% of crop removal in yield. Below is a detailed description of the new fertilizer allocation process using the Northeast (Region 1) and the Corn Belt (Region 5) as examples. The step-by-step allocations used trial and error fitting to crop needs and available fertilizer and manure data. P needs met first because some P fertilizers are combined with N such as manure and Diammonium Phosphate (DAP) which means N needs are then jointly met. Region 1 has nearly double the amount of phosphorus available in fertilizer and manure as removed by Agricultural Census crop yields estimates. This is probably due to much of the fertilizer being applied to crops not accounted for by the crops we are simulating or non-farm crops like lawns, golf courses, orchards, etc. See example step by step allocations below.

Northeast (Region 1) Fertilizer Allocation Process Steps as a % of Crop Needs

Phosphorus Manure (see Table S4, color coded)

1. Allocate dairy manure to corn silage 100 % spring before plant
2. Allocate dairy manure to sorghum silage 100 % spring before plant
3. Allocate dairy manure to Hay 70% spring before plant
4. Allocate dairy manure to Alfalfa 100% spring before plant
5. Allocate dairy manure to other grass 80% spring before plant
6. Allocate dairy manure to Corn grain 60% spring before plant
7. Allocate dairy manure to Sorghum Grain 50% spring before plant
8. Allocate dairy manure to Winter Wheat 50% spring after fall plant
9. Allocate poultry manure to Hay 30% spring before plant
10. Allocate poultry manure to other grass 20% spring before plant
11. Allocate swine manure to corn grain 35% spring before plant
12. Allocate Beef manure to corn grain 5% spring before plant

Phosphorus (see Table S4, color coded)

13. Allocate P identified by grade to meet cotton needs 100% spring at plant
14. Allocate P identified by grade to meet peanut needs 100% spring before plant
15. Allocate P identified by grade to meet rice needs 100% spring before plant
16. Allocate P identified by grade to meet potatoes needs 100% spring before plant
17. Allocate P identified by grade to meet other crop needs 100% spring before plant
18. Allocate P identified by grade to meet canola needs 100% spring at plant
19. Allocate P identified by grade to meet peas needs 100% spring before plant
20. Allocate P identified by grade to meet Barley needs 50% spring before plant
21. Allocate P identified by grade to meet edible beans needs 50% spring before plant
22. Allocate P identified by grade to meet oats needs 50% spring before plant
23. Allocate P identified by grade to meet sorghum grain needs 50% spring before plant
24. Allocate P identified by grade to meet soybeans needs 50% spring before plant
25. Allocate P identified by grade to meet spring wheat needs 50% spring before plant
26. Allocate P identified by grade to meet rye needs 100% fall before plant

27. Allocate Mono ammonium phos to meet Barley needs 50% spring before plant
28. Allocate Mono ammonium phos to meet edible beans needs 50% spring before plant
29. Allocate Mono ammonium phos to meet oats needs 50% spring before plant
30. Allocate Mono ammonium phos to meet spring wheat needs 50% spring before plant
31. Allocate Mono ammonium phos to meet soybeans needs 25% spring before plant
32. Allocate DAP to meet Soybeans needs 15% spring before plant
33. Allocate DAP to meet Winter Wheat needs 30% fall before plant
34. Allocate DAP to meet Winter Wheat needs 20% spring fall plant
35. Allocate liquid ammonium poly to meet soybeans needs 10% spring before plant

Nitrogen (see Table S4, color coded)

1. Allocate N identified by grade to meet cotton needs 100% spring at plant
2. Allocate N identified by grade to meet peanut needs 100% spring before plant
3. Allocate N identified by grade to meet rice needs 100% spring before plant
4. Allocate N identified by grade to meet potatoes needs 100% spring before plant
5. Allocate N identified by grade to meet other crop needs 100% spring before plant
6. Allocate N identified by grade to meet canola needs 100% spring before plant
7. Allocate N identified by grade to meet peas needs 100% spring before plant
8. Allocate 30% nitrogen to meet corn silage needs 74% at plant
9. Allocate 30% nitrogen to meet corn silage needs 15% after plant
10. Allocate 32% nitrogen to meet corn grain needs 19% at plant
11. Allocate 32% nitrogen to meet corn grain needs 4% after plant
12. Allocate 28% nitrogen to meet corn grain needs 6% at plant
13. Allocate 28% nitrogen to meet corn grain needs 2% after plant
14. Allocate N identified by grade to meet corn grain needs 40% in spring before plant
15. Allocate N identified by grade to meet corn grain needs 10% after plant
16. Allocate Anhydrous ammonia to meet Rye needs 100% in fall before plant
17. Allocate urea to meet nitrogen needs for sorghum grain needs 94% spring before plant
18. Allocate urea to meet nitrogen needs for sorghum silage needs 89% spring before plant
19. Allocate urea to meet nitrogen needs for winter wheat needs 88% spring with fall plant
20. Allocate urea to meet nitrogen needs for spring wheat needs 97% spring before plant
21. Allocate urea to meet nitrogen needs for barley needs 95% spring before plant
22. Allocate urea to meet nitrogen needs for oats needs 95% spring before plant
23. Allocate urea to meet nitrogen needs for hay needs 5% spring before plant
24. Allocate urea to meet nitrogen needs for hay needs 5% after plant
25. Allocate ammonium sulfate to meet nitrogen needs for hay needs 10% spring before plant
26. Allocate ammonium sulfate to meet nitrogen needs for hay needs 2% after plant
27. Allocate N identified by grade to meet hay needs 59% in spring before plant
28. Allocate N identified by grade to meet other grass needs 86% in spring before plant

Table S4: Northeast (Region 1) fertilizer allocation.

Regional file name [a]		IBELD4CROP odd# rainfed; (even# irrigated not listed)	FertSales ID	FertSalesName	FertNumEPIC + Timing	Timing ID	FertFrac	FertFracN	FertFracP
REGION01.C22	REGION01.C23	1Hay	22	N BY GRADE elem N	22 S B4 plt	4	0.59	0.59	0
REGION01.C22	REGION01.C23	1Hay	24	AMMONIUM SULFATE	32 S B4 plt	4	0.1	0.1	0
REGION01.C22	REGION01.C23	1Hay	66	UREA	51 S B4 plt	4	0.05	0.05	0
REGION01.C22	REGION01.C23	1Hay	118	CEAP118: Dairy manure	118 S B4 plt	4	0.7	0	0.7
REGION01.C22	REGION01.C23	1Hay	136	CEAP136: poultry manure	136 S B4 plt	4	0.3	0	0.3
REGION01.C22	REGION01.C23	1Hay	24	AMMONIUM SULFATE	32 S After p	6	0.02	0.02	0
REGION01.C22	REGION01.C23	1Hay	66	UREA	51 S After p	6	0.05	0.05	0
REGION01.C24	REGION01.C25	3Alfalfa	118	CEAP118: Dairy manure	118 S B4 plt	4	1	0	1
REGION01.C26	REGION01.C27	5Other_Gra	22	N BY GRADE elem N	22 S B4 plt	4	0.86	0.86	0
REGION01.C26	REGION01.C27	5Other_Gra	118	CEAP118: Dairy manure	118 S B4 plt	4	0.8	0	0.8
REGION01.C26	REGION01.C27	5Other_Gra	136	CEAP136: poultry manure	136 S B4 plt	4	0.2	0	0.2
REGION01.C28	REGION01.C29	7Barley	23	P2O5 BY GRADE elem P	23 S B4 plt	4	0.5	0	0.5
REGION01.C28	REGION01.C29	7Barley	66	UREA	51 S B4 plt	4	0.95	0.95	0
REGION01.C28	REGION01.C29	7Barley	209	MONOAMM PHOS	64 S B4 plt	4	0.5	0	0.5
REGION01.C30	REGION01.C31	9BeansEdib	23	P2O5 BY GRADE elem P	23 S B4 plt	4	0.5	0	0.5
REGION01.C30	REGION01.C31	9BeansEdib	209	MONOAMM PHOS	64 S B4 plt	4	0.5	0	0.5
REGION01.C32	REGION01.C33	11CornGrain	22	N BY GRADE elem N	22 S B4 plt	4	0.4	0.4	0
REGION01.C32	REGION01.C33	11CornGrain	104	CEAP104: Beef Solids	104 S B4 plt	4	0.05	0	0.05
REGION01.C32	REGION01.C33	11CornGrain	118	CEAP118: Dairy manure	118 S B4 plt	4	0.6	0	0.6
REGION01.C32	REGION01.C33	11CornGrain	129	CEAP129: Swine solids all	129 S B4 plt	4	0.35	0	0.35
REGION01.C32	REGION01.C33	11CornGrain	58	NITROGEN SOL 28%	45 S At Plan	5	0.06	0.06	0
REGION01.C32	REGION01.C33	11CornGrain	60	NITROGEN SOL 32%	47 S At Plan	5	0.19	0.19	0
REGION01.C32	REGION01.C33	11CornGrain	22	N BY GRADE elem N	22 S After p	6	0.1	0.1	0
REGION01.C32	REGION01.C33	11CornGrain	58	NITROGEN SOL 28%	45 S After p	6	0.02	0.02	0
REGION01.C32	REGION01.C33	11CornGrain	60	NITROGEN SOL 32%	47 S After p	6	0.04	0.04	0
REGION01.C34	REGION01.C35	13CornSilag	118	CEAP118: Dairy manure	118 S B4 plt	4	1	0	1
REGION01.C34	REGION01.C35	13CornSilag	59	NITROGEN SOL 30%	46 S At Plan	5	0.74	0.74	0
REGION01.C34	REGION01.C35	13CornSilag	59	NITROGEN SOL 30%	46 S After p	6	0.15	0.15	0
REGION01.C36	REGION01.C37	15Cotton	22	N BY GRADE elem N	22 S At Plan	5	1	1	0
REGION01.C36	REGION01.C37	15Cotton	23	P2O5 BY GRADE elem P	23 S At Plan	5	1	0	1
REGION01.C38	REGION01.C39	17Oats	23	P2O5 BY GRADE elem P	23 S B4 plt	4	0.5	0	0.5
REGION01.C38	REGION01.C39	17Oats	66	UREA	51 S B4 plt	4	0.95	0.95	0
REGION01.C38	REGION01.C39	17Oats	209	MONOAMM PHOS	64 S B4 plt	4	0.5	0	0.5
REGION01.C40	REGION01.C41	19Peanuts	22	N BY GRADE elem N	22 S B4 plt	4	1	1	0
REGION01.C40	REGION01.C41	19Peanuts	23	P2O5 BY GRADE elem P	23 S B4 plt	4	1	0	1
REGION01.C42	REGION01.C43	21Potatoes	22	N BY GRADE elem N	22 S B4 plt	4	1	1	0

REGION01.C42	REGION01.C43	21Potatoes	23	P2O5 BY GRADE elem P	23 S B4 plt	4	1	0	1
REGION01.C44	REGION01.C45	23Rice	22	N BY GRADE elem N	22 S B4 plt	4	1	1	0
REGION01.C44	REGION01.C45	23Rice	23	P2O5 BY GRADE elem P	23 S B4 plt	4	1	0	1
REGION01.C46	REGION01.C47	25Rye	23	P2O5 BY GRADE elem P	23 F-B4 plt&	2	1	0	1
REGION01.C46	REGION01.C47	25Rye	2	ANHY AMMONIA	25 F-B4 plt&	2	1	1	0
REGION01.C48	REGION01.C49	27SorghumGr	23	P2O5 BY GRADE elem P	23 S B4 plt	4	0.5	0	0.5
REGION01.C48	REGION01.C49	27SorghumGr	66	UREA	51 S B4 plt	4	0.94	0.94	0
REGION01.C48	REGION01.C49	27SorghumGr	118	CEAP118: Dairy manure	118 S B4 plt	4	0.5	0	0.5
REGION01.C50	REGION01.C51	29SorghumSi	66	UREA	51 S B4 plt	4	0.89	0.89	0
REGION01.C50	REGION01.C51	29SorghumSi	118	CEAP118: Dairy manure	118 S B4 plt	4	1	0	1
REGION01.C52	REGION01.C53	31Soybeans	23	P2O5 BY GRADE elem P	23 S B4 plt	4	0.5	0	0.5
REGION01.C52	REGION01.C53	31Soybeans	203	DAP	58 S B4 plt	4	0.15	0	0.15
REGION01.C52	REGION01.C53	31Soybeans	209	MONOAMM PHOS	64 S B4 plt	4	0.25	0	0.25
REGION01.C52	REGION01.C53	31Soybeans	249	LIQ AMM POLY	76 S B4 plt	4	0.1	0	0.1
REGION01.C54	REGION01.C55	33Wheat_Spr	23	P2O5 BY GRADE elem P	23 S B4 plt	4	0.5	0	0.5
REGION01.C54	REGION01.C55	33Wheat_Spr	66	UREA	51 S B4 plt	4	0.97	0.97	0
REGION01.C54	REGION01.C55	33Wheat_Spr	209	MONOAMM PHOS	64 S B4 plt	4	0.5	0	0.5
REGION01.C56	REGION01.C57	35Wheat_Win	203	DAP	58 F-B4 plt&	2	0.3	0	0.3
REGION01.C56	REGION01.C57	35Wheat_Win	66	UREA	51 S with Fa	3	0.88	0.88	0
REGION01.C56	REGION01.C57	35Wheat_Win	203	DAP	58 S with Fa	3	0.2	0	0.2
REGION01.C56	REGION01.C57	35Wheat_Win	118	CEAP118: Dairy manure	118 S with Fa	3	0.5	0	0.5
REGION01.C58	REGION01.C59	37Other_Cro	22	N BY GRADE elem N	22 S B4 plt	4	1	1	0
REGION01.C58	REGION01.C59	37Other_Cro	23	P2O5 BY GRADE elem P	23 S B4 plt	4	1	0	1
REGION01.C60	REGION01.C61	39Canola	22	N BY GRADE elem N	22 S At Plan	5	1	1	0
REGION01.C60	REGION01.C61	39Canola	23	P2O5 BY GRADE elem P	23 S At Plan	5	1	0	1
REGION01.C62	REGION01.C63	41Peas	22	N BY GRADE elem N	22 S B4 plt	4	1	1	0
REGION01.C62	REGION01.C63	41Peas	23	P2O5 BY GRADE elem P	23 S B4 plt	4	1	0	1

[a]: The data source files for fertilizer types, timing of application, and fraction of allocation for each crop in each of the 10 production regions were organized as file name REGION*.C# (saved under …\Festc1.4.1\Epic\common_data\util\manageCreate\regionFert\ REGION*.C#), where * is the production Region ID number ranging from 01 to 10, and # is the "Crop Code" from 22 to 63 as listed in Table S2.

Corn Belt (Region 5) Fertilizer Allocation Process Steps as a % of Crop Needs

Phosphorus Manure (see Table S5, color coded)

1. Allocate dairy manure to corn silage 100 % spring before plant
2. Allocate dairy manure to sorghum silage 100 % spring before plant
3. Allocate dairy manure to Hay 80% spring before plant
4. Allocate dairy manure to Alfalfa 100% spring before plant
5. Allocate dairy manure to other grass 85% spring before plant
6. Allocate poultry manure to corn grain 5% spring before plant
7. Allocate swine manure to corn grain 37.5% spring before plant
8. Allocate Beef manure to other grass 15% spring before plant
9. Allocate Beef manure to Hay 20% spring before plant

Phosphorus (see Table S5, color coded)

10. Allocate P identified by grade to meet <u>other crop</u> needs 100% spring before plant
11. Allocate P identified by grade to meet <u>canola</u> needs 100% spring before plant
12. Allocate P identified by grade to meet <u>peas</u> needs 100% spring before plant
13. Allocate P identified by grade to meet <u>soybean</u> needs 20% spring before plant
14. Allocate P identified by grade to meet <u>soybean</u> needs 15% fall before and at plant
15. Allocate Mono ammonium phos to meet <u>soybean</u> needs 30% spring before plant
16. Allocate DAP to meet <u>Soybean</u> needs 15% spring before plant
17. Allocate DAP to meet <u>Winter Wheat</u> needs 100% fall before plant
18. Allocate DAP to meet <u>Spring Wheat</u> needs 100% fall before plant
19. Allocate DAP to meet <u>Barley</u> needs 100% fall before plant
20. Allocate DAP to meet <u>edible beans</u> needs 100% spring before plant
21. Allocate DAP to meet <u>Corn Grain</u> needs 50% fall before plant
22. Allocate DAP to meet <u>Corn Grain</u> needs 7.5% spring before plant
23. Allocate DAP to meet <u>Cotton</u> needs 100% spring before plant
24. Allocate DAP to meet <u>oats</u> needs 100% spring before plant
25. Allocate DAP to meet <u>peanuts</u> needs 100% spring before plant
26. Allocate DAP to meet <u>potatoes</u> needs 100% spring before plant
27. Allocate DAP to meet <u>Rice</u> needs 100% spring before plant
28. Allocate DAP to meet <u>Rye</u> needs 100% fall before plant
29. Allocate DAP to meet <u>sorghum grain</u> needs 100% spring before plant
30. Allocate liquid ammonium poly to meet <u>soybeans</u> needs 10% spring before plant
31. Allocate liquid ammonium poly to meet <u>soybeans</u> needs 10% fall before and at plant

Nitrogen (see Table S5, color coded)

29. Allocate N identified by grade to meet hay needs 70% spring before plant
30. Allocate N identified by grade to meet hay needs 25% spring after plant
31. Allocate N identified by grade to meet other crop needs 10% spring after plant
32. Allocate N identified by grade to meet Alfalfa needs 10% spring before plant
33. Allocate 32% nitrogen to meet corn grain needs 20% at plant
34. Allocate 32% nitrogen to meet corn grain needs 5% after plant
35. Allocate 28% nitrogen to meet corn grain needs 6% after plant
36. Allocate urea to meet nitrogen needs for other crop needs 100% spring before plant
37. Allocate urea to meet nitrogen needs for sorghum grain needs 85% spring before plant
38. Allocate urea to meet nitrogen needs for sorghum silage needs 89% spring before plant
39. Allocate urea to meet nitrogen needs for winter wheat needs 12% spring with fall plant
40. Allocate urea to meet nitrogen needs for spring wheat needs 88% spring before plant
41. Allocate urea to meet nitrogen needs for barley needs 81% spring before plant
42. Allocate urea to meet nitrogen needs for oats needs 82% spring before plant
43. Allocate urea to meet nitrogen needs for Canola needs 10% spring before plant
44. Allocate urea to meet nitrogen needs for Peas needs 10% spring before plant
45. Allocate urea to meet nitrogen needs for Rye needs 70% fall before plant
46. Allocate urea to meet nitrogen needs for Rye needs 14% spring with plant

47. Allocate urea to meet nitrogen needs for Rice needs 60% spring before plant
48. Allocate urea to meet nitrogen needs for Rice needs 27% spring after plant
49. Allocate urea to meet nitrogen needs for potatoes needs 82% spring before plant
50. Allocate urea to meet nitrogen needs for Cotton needs 87% spring before plant
51. Allocate urea to meet nitrogen needs for Corn silage needs 89% spring before plant
52. Allocate urea to meet nitrogen needs for other grass needs 86% spring before plant
53. Allocate ammonium sulfate to meet nitrogen needs for corn grain needs 50% fall before plant
54. Allocate ammonium sulfate to meet nitrogen needs for winter wheat needs 15% fall before plant
55. Allocate ammonium sulfate to meet nitrogen needs for winter wheat needs 60% at fall plant

Table S5: Corn Belt (Region 5) fertilizer allocation.

Regional file name [a]		IBELD4CROP odd# rainfed; (even# irrigated not listed)	FertSales ID	FertSalesName	FertNumEPIC + Timing	Timing ID	FertFrac	FertFracN	FertFracP
REGION05.C22	REGION05.C23	1Hay	22	N BY GRADE elem N	22 S B4 plt	4	0.7	0.7	0
REGION05.C22	REGION05.C23	1Hay	104	CEAP104: Beef Solids	104 S B4 plt	4	0.2	0	0.2
REGION05.C22	REGION05.C23	1Hay	118	CEAP118: Dairy manure	118 S B4 plt	4	0.8	0	0.8
REGION05.C22	REGION05.C23	1Hay	22	N BY GRADE elem N	22 S After p	6	0.25	0.25	0
REGION05.C24	REGION05.C25	3Alfalfa	22	N BY GRADE elem N	22 S B4 plt	4	0.1	0.1	0
REGION05.C24	REGION05.C25	3Alfalfa	118	CEAP118: Dairy manure	118 S B4 plt	4	1	0	1
REGION05.C26	REGION05.C27	5Other_Gra	66	UREA	51 S B4 plt	4	0.86	0.86	0
REGION05.C26	REGION05.C27	5Other_Gra	104	CEAP104: Beef Solids	104 S B4 plt	4	0.15	0	0.15
REGION05.C26	REGION05.C27	5Other_Gra	118	CEAP118: Dairy manure	118 S B4 plt	4	0.85	0	0.85
REGION05.C26	REGION05.C27	5Other_Gra	22	N BY GRADE elem N	22 S After p	6	0.1	0.1	0
REGION05.C28	REGION05.C29	7Barley	203	DAP	58 F-B4 plt&	2	1	0	1
REGION05.C28	REGION05.C29	7Barley	66	UREA	51 S B4 plt	4	0.81	0.81	0
REGION05.C30	REGION05.C31	9BeansEdib	203	DAP	58 S B4 plt	4	1	0	1
REGION05.C32	REGION05.C33	11CornGrain	2	ANHY AMMONIA	25 F-B4 plt&	2	0.5	0.5	0
REGION05.C32	REGION05.C33	11CornGrain	203	DAP	58 F-B4 plt&	2	0.5	0	0.5
REGION05.C32	REGION05.C33	11CornGrain	203	DAP	58 S B4 plt	4	0.075	0	0.075
REGION05.C32	REGION05.C33	11CornGrain	129	CEAP129: Swine solids all	129 S B4 plt	4	0.375	0	0.375
REGION05.C32	REGION05.C33	11CornGrain	136	CEAP136: poultry manure	136 S B4 plt	4	0.05	0	0.05
REGION05.C32	REGION05.C33	11CornGrain	60	NITROGEN SOL 32%	47 S At Plan	5	0.2	0.2	0
REGION05.C32	REGION05.C33	11CornGrain	58	NITROGEN SOL 28%	45 S After p	6	0.06	0.06	0
REGION05.C32	REGION05.C32	11CornGrain	60	NITROGEN SOL 32%	47 S After p	6	0.05	0.05	0
REGION05.C34	REGION05.C34	13CornSilag	66	UREA	51 S B4 plt	4	0.89	0.89	0
REGION05.C34	REGION05.C34	13CornSilag	118	CEAP118: Dairy manure	118 S B4 plt	4	1	0	1
REGION05.C36	REGION05.C36	15Cotton	66	UREA	51 S B4 plt	4	0.87	0.87	0
REGION05.C36	REGION05.C36	15Cotton	203	DAP	58 S B4 plt	4	1	0	1
REGION05.C38	REGION05.C38	17Oats	66	UREA	51 S B4 plt	4	0.82	0.82	0
REGION05.C38	REGION05.C38	17Oats	203	DAP	58 S B4 plt	4	1	0	1
REGION05.C40	REGION05.C40	19Peanuts	203	DAP	58 S B4 plt	4	1	0	1

REGION05.C42	REGION05.C42	21Potatoes	66	UREA	51 S B4 plt	4	0.82	0.82	0
REGION05.C42	REGION05.C42	21Potatoes	203	DAP	58 S B4 plt	4	1	0	1
REGION05.C44	REGION05.C44	23Rice	66	UREA	51 S B4 plt	4	0.6	0.6	0
REGION05.C44	REGION05.C44	23Rice	203	DAP	58 S B4 plt	4	1	0	1
REGION05.C44	REGION05.C44	23Rice	66	UREA	51 S After p	6	0.27	0.27	0
REGION05.C46	REGION05.C46	25Rye	66	UREA	51 F-B4 plt&	2	0.7	0.7	0
REGION05.C46	REGION05.C46	25Rye	203	DAP	58 F-B4 plt&	2	1	0	1
REGION05.C46	REGION05.C46	25Rye	66	UREA	51 S with Fa	3	0.14	0.14	0
REGION05.C48	REGION05.C48	27SorghumGr	66	UREA	51 S B4 plt	4	0.85	0.85	0
REGION05.C48	REGION05.C48	27SorghumGr	203	DAP	58 S B4 plt	4	1	0	1
REGION05.C50	REGION05.C50	29SorghumSi	66	UREA	51 S B4 plt	4	0.89	0.89	0
REGION05.C50	REGION05.C50	29SorghumSi	118	CEAP118: Dairy manure	118 S B4 plt	4	1	0	1
REGION05.C52	REGION05.C52	31Soybeans	23	P2O5 BY GRADE elem P	23 F-B4 plt&	2	0.15	0	0.15
REGION05.C52	REGION05.C52	31Soybeans	249	LIQ AMM POLY	76 F-B4 plt&	2	0.1	0	0.1
REGION05.C52	REGION05.C52	31Soybeans	23	P2O5 BY GRADE elem P	23 S B4 plt	4	0.2	0	0.2
REGION05.C52	REGION05.C52	31Soybeans	203	DAP	58 S B4 plt	4	0.15	0	0.15
REGION05.C52	REGION05.C52	31Soybeans	209	MONOAMM PHOS	64 S B4 plt	4	0.3	0	0.3
REGION05.C52	REGION05.C52	31Soybeans	249	LIQ AMM POLY	76 S B4 plt	4	0.1	0	0.1
REGION05.C54	REGION05.C54	33Wheat_Spr	203	DAP	58 F-B4 plt&	2	1	0	1
REGION05.C54	REGION05.C54	33Wheat_Spr	66	UREA	51 S B4 plt	4	0.88	0.88	0
REGION05.C56	REGION05.C56	35Wheat_Win	10	AMMONIUM NITRATE	27 F-B4 plt&	2	0.15	0.15	0
REGION05.C56	REGION05.C56	35Wheat_Win	203	DAP	58 F-B4 plt&	2	1	0	1
REGION05.C56	REGION05.C56	35Wheat_Win	10	AMMONIUM NITRATE	27 S with Fa	3	0.6	0.6	0
REGION05.C56	REGION05.C56	35Wheat_Win	66	UREA	51 S with Fa	3	0.12	0.12	0
REGION05.C58	REGION05.C58	37Other_Cro	23	P2O5 BY GRADE elem P	23 S B4 plt	4	1	0	1
REGION05.C58	REGION05.C58	37Other_Cro	66	UREA	51 S B4 plt	4	1	1	0
REGION05.C60	REGION05.C60	39Canola	23	P2O5 BY GRADE elem P	23 S B4 plt	4	1	0	1
REGION05.C60	REGION05.C60	39Canola	66	UREA	51 S B4 plt	4	0.1	0.1	0
REGION05.C62	REGION05.C62	41Peas	23	P2O5 BY GRADE elem P	23 S B4 plt	4	1	0	1
REGION05.C62	REGION05.C62	41Peas	66	UREA	51 S B4 plt	4	0.1	0.1	0

[a]: The data source files for fertilizer types, timing of application, and fraction of allocation for each crop in each of the 10 production regions were organized as file name REGION*.C# (saved under …\Festc1.4.1\Epic\common_data\util\manageCreate\regionFert\ REGION*.C#), where * is the production Region ID number ranging from 01 to 10, and # is the "Crop Code" from 22 to 63 as listed in Table S2.

The allocation process is conducted within a set of linked spreadsheets to give the person making the allocation instant feedback as the allocation process proceeds. There is no data base to check the allocation other than the fertilizer sales which are not identified by crop fertilized. There is always the possibility to adjust if the fertilizer industry or agricultural producers provide guidance.

2.3.3. Fertilizer application rate

In most cases, the majority of N is applied immediately before or at crop planting. For each crop in the US, Goebes et al. (2003) assigned a fixed pre-plant allocation, applied during a fixed window, lasting several weeks to 2 months, across all simulation years [56]. For this modeling

system, which is to automate to the greatest extent possible, the generation of fertilizer management for each 12 km by 12 km grid and crop, the amount of each fertilizer (2006 FMS) and/or manure application (2011 FMS) for each crop is based on annual EPIC 5-yr climatological average amount from the last 5-yr of the 25 years spin-up runs. The spin-up estimate of fertilizer N need is based on stress triggered applications and application amount is the percentage of total N need. A 10% adjustment was incorporated considering runoff loss based on CEAP. The elemental N estimate is then converted into fertilizer amount for each fertilizer by timing by calculating the amount needed to supply the N needs from the spin-up simulations.

For both FMS development, for each 12 km by 12 km grid and crop, the amount of N initially applied is a fixed fraction of an annual EPIC 5-yr climatological average amount, but the date of application will vary with crop, crop variety, local soil and weather conditions leading to more spatially and temporally resolved application estimates. The N form dictates the equipment used to apply the fertilizer, the depth of application and application timing, which in turn affects subsequent volatilization and other biogeochemical process rates as well as surface and sub-surface losses. The fraction-of-annual-total for each fertilizer form is distributed to meet crop N demand in a production region, based on documented crop management practices and yield value. For example, more costly N forms are assigned to higher-value crops. For the 2006 FMS, the fertilizer sales data from USDA-NASS for 2006 were allocated for crop use. When crop demand exceeds inorganic agricultural N sales, the shortfall is assumed to be met with manure [18]. The application presented in [18] reflects market conditions for a base year, 2002. However, for the 2011 FMS, each fertilizer from fertilizer sales or manure were used for fertilizer allocation (Tables S3-S5).
